# Neurofilaments in health and Charcot-Marie-Tooth disease

**DOI:** 10.3389/fcell.2023.1275155

**Published:** 2023-12-18

**Authors:** Farah Kotaich, Damien Caillol, Pascale Bomont

**Affiliations:** ERC team, NeuroMyoGene Institute-Pathophysiology and Genetics of Neuron and Muscle, Inserm U1315, CNRS UMR5261, University of Lyon 1, Lyon, France

**Keywords:** neurofilament, cytoskeleton, intermediate filaments, Charcot-Marie-Tooth disease, CMT, neurodegenerative diseases, mutations, dynamics

## Abstract

Neurofilaments (NFs) are the most abundant component of mature neurons, that interconnect with actin and microtubules to form the cytoskeleton. Specifically expressed in the nervous system, NFs present the particularity within the Intermediate Filament family of being formed by four subunits, the neurofilament light (NF-L), medium (NF-M), heavy (NF-H) proteins and α-internexin or peripherin. Here, we review the current knowledge on NF proteins and neurofilaments, from their domain structures and their model of assembly to the dynamics of their transport and degradation along the axon. The formation of the filament and its behaviour are regulated by various determinants, including post-transcriptional (miRNA and RBP proteins) and post-translational (phosphorylation and ubiquitination) modifiers. Altogether, the complex set of modifications enable the neuron to establish a stable but elastic NF array constituting the structural scaffold of the axon, while permitting the local expression of NF proteins and providing the dynamics necessary to fulfil local demands and respond to stimuli and injury. Thus, in addition to their roles in mechano-resistance, radial axonal outgrowth and nerve conduction, NFs control microtubule dynamics, organelle distribution and neurotransmission at the synapse. We discuss how the studies of neurodegenerative diseases with NF aggregation shed light on the biology of NFs. In particular, the *NEFL* and *NEFH* genes are mutated in Charcot-Marie-Tooth (CMT) disease, the most common inherited neurological disorder of the peripheral nervous system. The clinical features of the CMT forms (axonal CMT2E, CMT2CC; demyelinating CMT1F; intermediate I-CMT) with symptoms affecting the central nervous system (CNS) will allow us to further investigate the physiological roles of NFs in the brain. Thus, NF-CMT mouse models exhibit various degrees of sensory-motor deficits associated with CNS symptoms. Cellular systems brought findings regarding the dominant effect of NF-L mutants on NF aggregation and transport, although these have been recently challenged. Neurofilament detection without NF-L in recessive CMT is puzzling, calling for a re-examination of the current model in which NF-L is indispensable for NF assembly. Overall, we discuss how the fundamental and translational fields are feeding each-other to increase but also challenge our knowledge of NF biology, and to develop therapeutic avenues for CMT and neurodegenerative diseases with NF aggregation.

## 1 Introduction

Neurofilaments (NFs) belong to a family of proteins called Intermediate Filaments (IFs), that form the cytoskeleton of the cell together with the actin filaments and microtubules (MTs) (Eriksson et al., 2009; Herrmann et al., 2009). The name refers to the 10 nm diameter of the filaments that is categorised as “intermediate” between the 7 nm actin filaments (Grazi et al., 1993) and 25 nm MT (Wade, 2009). IF proteins are encoded by one of the largest gene families present in the human genome that comprises ∼70 genes and phylogenetically derives from a common lamin-like ancestor (Peter and Stick, 2015). Historically, the classification of IFs was divided into 6 major groups according to their amino acid sequences and their structural and physico-chemical properties: types I and II are constituted by acidic and neutral-basic keratin proteins, respectively; type III includes vimentin, desmin, GFAP and peripherin; type IV comprises nestin, synemin, ⍺-internexin and NF proteins; type V is composed of the nuclear lamins and type VI by the lens-specific Bfsp1/Bfsp2 (Eriksson et al., 2009; Omary, 2009). Gene expression of IFs has been shown to be tissue-specific (Omary, 2009) to provide a customizable cytoplasmic meshwork adapted to various tissues and cell types. Thus, mutations in *IF* genes have been shown to cause a wide range of >80 tissue-specific diseases, from skin diseases (keratin), myopathies (desmin), cataract (Bfsp) to neuropathies (NFs, GFAP) (Omary, 2009). The crucial roles of IFs in physiology is further supported by the discovery of a wide aggregation of IF proteins in numerous pathologies, including desmin bodies in myopathies and NF accumulation in most neurodegenerative diseases (Omary, 2009). Within the IF family, NFs are selectively expressed in the nervous system, where they constitute the most abundant cytoskeletal component of mature neurons and represent as much as 15% of the total protein content of peripheral nerves (Yuan et al., 2017). NFs form a backbone structure composed of three subunits - the neurofilament light protein (NF-L), the neurofilament medium protein (NF-M) and the neurofilament heavy protein (NF-H). This structure can further incorporate ⍺-internexin in the central nervous system (CNS) and peripherin in the peripheral nervous system (Liem and Messing, 2009). The manipulation of NF stoichiometry in mice has revealed the fundamental role of NFs in the development, maintenance, survival and regeneration of the nervous system (Lariviere and Julien, 2004; Liem and Messing, 2009). In the biomedical field, NFs have received much attention as their aggregation constitutes a pathological hallmark for most neurodegenerative diseases (Liem and Messing, 2009; Perrot and Eyer, 2009; Didonna and Opal, 2019) and they represent a universal biomarker for neurodegeneration (Gafson et al., 2020; Bomont, 2021), as a result of their detection in cerebrospinal fluid and blood. While the exact contribution of NF aggregation in disease still remains unclear, the discovery of disease-causing mutations in genes controlling the dynamics of NFs (*GAN*, *TRIM2*, *KIF5A*, *SACS*, *HSPB1*) further evidence their importance for neuronal homeostasis and functions (Bomont, 2021). More directly, the *NEFL* and *NEFH* genes have been shown to be mutated in several forms of the Charcot-Marie-Tooth (CMT) disease (Laurá et al., 2019), the most common inherited neurological disorder of the peripheral nervous system.

This review aims to present the achievements of both fields of fundamental research and study of diseases and to discuss how these findings have enriched our knowledge of NF biology and roles in physiology. First, we will present NFs, from their assembly to their dynamics and roles in neuronal physiology. Secondly, we will focus on diseases to detail the genetics of NF-CMTs, the cellular and physiological effects of NF mutants *in vitro* and in animal models, to reach conclusions on the ongoing therapeutic perspectives towards NFs.

## 2 The neurofilament network

### 2.1 Structural organisation and assembly of neurofilaments

**FIGURE 1:**
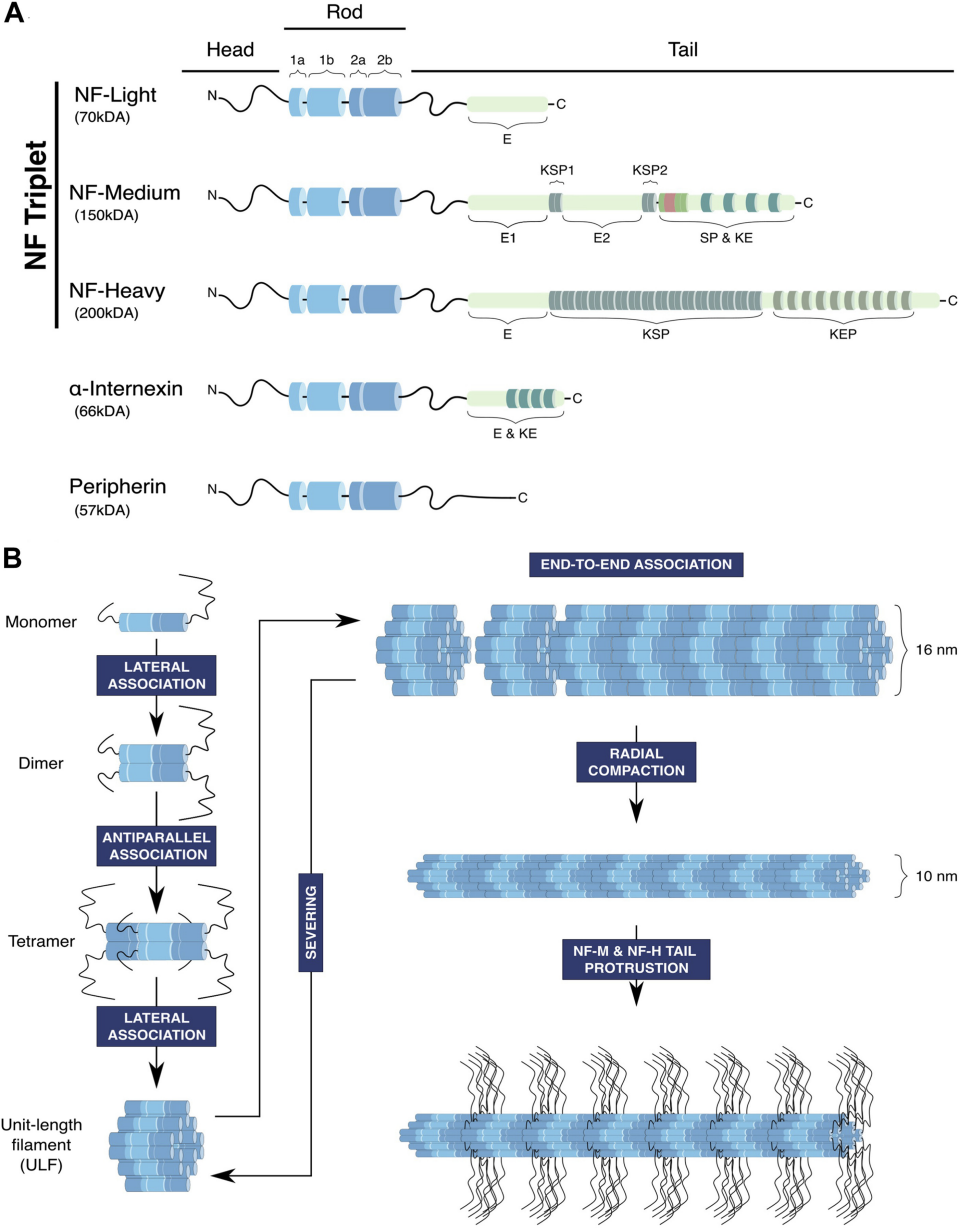
Structure and assembly of neurofilaments. **(A)** Neurofilaments are composed of the core NF triplet: NF-Light (NF-L), NF-Medium (NF-M) and NF-Heavy (NF-H) subunits, which integrates a fourth subunit, peripherin in the peripheral nervous system and ⍺-internexin in the central nervous system. NF subunits have a tripartite structure composed of a head domain, a conserved rod domain containing α-helical coils ensuring coiled-coil interactions necessary for NF assembly and a tail domain of variable length that is subjected to post-translational modifications, in particular phosphorylation of lysine-serin-proline repeats (KSP). (E: glutamic-acid-rich segment, KE: lysine-glutamic acid, KEP: lysine-glutamic acid-proline, SP: serine-proline, KSP: lysine-serine-proline). **(B)** NFs follow an assembly process common to other IFs, starting with a sequence of successive association of polypeptides to form the basic unit of NFs, the Unit-Length Filament (ULF). Firstly, the rod domains of NF monomers interact to form parallel coiled-coil heterodimers. Secondly, dimers associate and generate antiparallel tetramers, which consequently lose their polarity. Then, the lateral association of eight tetramers forms a cylindrical structure called the unit-length filament (ULF), which can expand through end-to-end association (annealing) to generate the neurofilament. In principle, fragmentation may occur through the severing of the filament, but whether this mechanism acts on the immature 16 nm filament is not determined. Finally, the filament matures through radial compaction to reach a diameter of 10 nm, and phosphorylation of the long tails of NF-M and NF-H induces their projection outward from the filament.

Like all members of the IF family, NFs have a tripartite structural organisation (Figure 1A). A central conserved segment consisting of an ⍺-helical rod domain is flanked by a short N-terminal segment named the head domain and a C-terminal segment of variable size named the tail domain (Herrmann and Aebi, 2016). In the IF family, NFs have the specificity of being heteropolymers formed by four proteins (Yuan et al., 2017): the NF triplet constituted by the NF-L (for Light), NF-M (for Medium) and NF-H (for Heavy) proteins, and a fourth subunit α-internexin or peripherin that is specifically expressed within the central and peripheral nervous system, respectively (Yuan et al., 2006; 2012). The rod domain contains long stretches of hydrophobic heptad repeats and promotes the assembly of the filament through the formation of ⍺-helical coiled-coil dimers (Herrmann and Aebi, 2016). Both extremities of the NF proteins can be subjected to various post-translational modifications to modulate the stability and organisation of the filament, in particular the long C-terminal domains of NF-M and NF-H which are highly phosphorylated.

With their specific composition, NFs are considered to assemble through a sequential mode similarly to other IF filaments (Figure 1B). The first step begins with the association of monomers to form parallel coiled-coil dimers though the central rod domain. Then, dimers associate in antiparallel tetramers, hence generating non-polar polymers. The subsequent step involves the lateral association of eight tetramers to form a ring structure called the Unit-Length Filaments (ULFs), which measures 16 nm in diameter (Herrmann et al., 1996). The ULF is thought to constitute the basic unit of NFs, whose longitudinal association through end-to-end annealing (and shortening through severing) will define the length of the filament (Colakoğlu and Brown, 2009; Uchida et al., 2013). Finally, NFs are subjected to radial compaction followed by the protrusion of the tail domains of the NF-M and NF-H subunits promoted by their phosphorylation, generating mature NFs of 10 nm in diameter able to interact with other components of the cytoskeleton and organelles. Indeed, ultrastructural studies have revealed that the regular projections radiating outward from the filament core (Hisanaga and Hirokawa, 1988) form cross-bridges between NFs, organelles and other cytoskeletal elements (Hirokawa, 1982).

Regarding the mode of assembly of the filament, *in vitro* studies revealed that NF assembly does not require nucleotide binding or hydrolysis but depends on the physicochemical properties of the subunits and the environment (temperature, pH, ions … ). While this supports the idea that the intrinsic nature of NF polypeptides can drive assembly, this does not exclude that cofactors can regulate their assembly *in vivo* (Chernyatina et al., 2015; Herrmann and Aebi, 2016). As examples, Sacsin (mutated in the ataxia ARSACs presenting NF aggregation), NUDEL and the 14-3-3 protein have been shown to bind to NF-L and regulate the assembly/disassembly state of NFs (Nguyen et al., 2004; Miao et al., 2013; Dabbaghizadeh et al., 2022). Investigation of the intrinsic capacities of single NF subunits to polymerise revealed that only NF-L, peripherin and α-internexin are able to form homopolymeric filaments *in vitro* (Ching and Liem, 1993; Cui et al., 1995; Carter et al., 1998). Thus, NF-M and NF-H incorporation require the presence of NF-L, supporting the notion that NF-L (together with peripherin or α-internexin) constitutes the backbone of the filament, exposing NF-M and NF-H (and their long tail domains) at the periphery. The assembly model would be that NF-L copolymerises with NF-M and NF-H, thus forming NF-L/NF-M and NF-L/NF-H hetero-tetramers, which are then able to associate together through NF-L to form the ULFs (Yuan et al., 2006; 2012).

### 2.2 Gene expression in development

**FIGURE 2:**
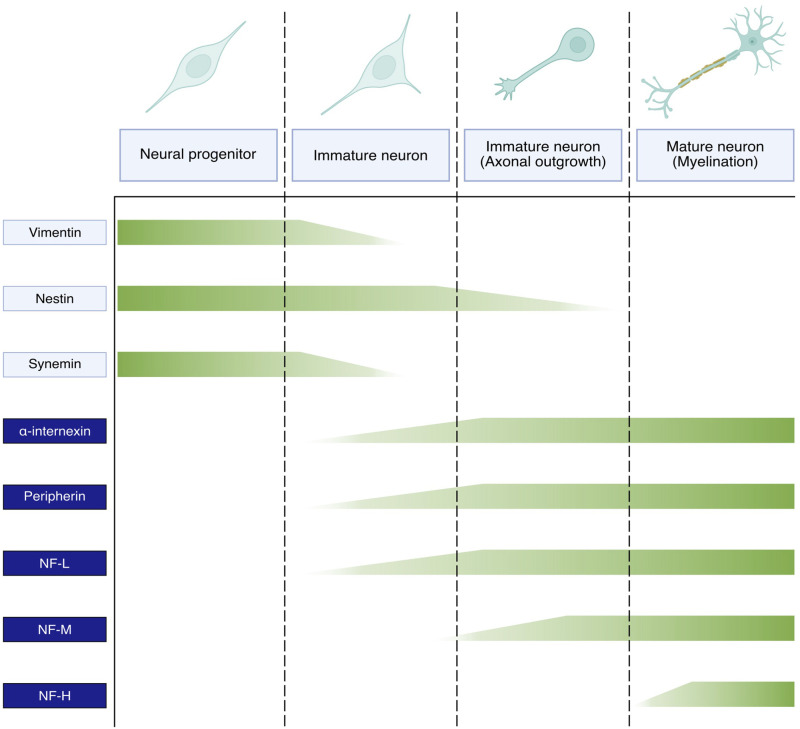
Expression pattern of neuronal intermediate filaments during neuronal maturation. Vimentin, nestin, synemin present in the ectodermal cells committed to neuronal differentiation are gradually downregulated in favour of NF subunits in immature neurons. The expression of NF proteins is sequential and coupled with neuronal maturation. Thus, NF-L alongside either ⍺-internexin in the CNS or peripherin in the PNS initially appear in immature neurons, followed by NF-M during axonal outgrowth and NF-H during axon myelination and radial outgrowth.

NFs are the most abundant cytoskeletal component of mature neurons and the expression of individual NF subunits follows a temporal sequence synchronised with neuronal development. There are discrepancies between different neuronal types, localisation in the brain and organism species, and consequently the exact expression profile of the different NF proteins is not fully established. Still, a general picture proposes that immature neurons do not express NF proteins but other IF types, such as vimentin, nestin and synemin (Bott and Winckler, 2020; Bomont, 2021). Upon neuronal differentiation, the expression of these IF types is downregulated and progressively replaced by NF proteins in a sequential manner (Figure 2). Thus, NF-L is first expressed (together with peripherin or α-internexin) upon neuronal differentiation, followed by NF-M during axonal elongation, and NF-H concomitantly to radial outgrowth and myelination (Kirkcaldie and Dwyer, 2017; Bomont, 2021). The subsequent phosphorylation of the NF-H and NF-M tails triggers the formation of cross-bridges between NF polymers, membranes and other cytoskeletal components, therefore stabilising the network and negatively regulating axonal elongation. The change in IF expression during neuronal maturation demonstrates the crucial role of NFs in neuronal maturation and physiology. Still, the concomitant and residual expression of several IF types in mature neurons suggests a cooperation of these different elements in sustaining neuronal functions, such as the presence of nestin in the distal region of growing axons (Bott and Winckler, 2020).

### 2.3 Post-transcriptional and post-translational modifications of neurofilaments

The ability of the cell to control gene expression and its proteome is essential for sustaining cell viability and physiological functions. These regulations can occur at the genomic, transcriptomic and protein levels. For NFs, both post-transcriptional and post-translational modifications (PTMs) have been identified.

Post-transcriptional regulations are RNA-mediated mechanisms that control many aspects of RNA life, including biogenesis, alternative splicing, stability, transport and translation efficiency. For NFs, post-transcriptional modifications have been found to be triggered by microRNAs (miRNAs) and RNA-binding proteins (RBPs) (Yuan and Nixon, 2023). Collectively, these regulatory mechanisms ensure a spatio-temporal expression of isoforms in specific neuronal compartments and are therefore essential for neuronal physiology. While miRNAs represent a common mechanism that is deregulated in neurodegenerative diseases (Juźwik et al., 2019), increasing evidence shows the direct role of RNA-mediated mechanisms in the pathophysiology of various neurodegenerative diseases, with dozens of RBP identified as causal genes for amyotrophic lateral sclerosis (ALS) and frontotemporal dementia (FTD) alone (Yuan and Nixon, 2023). Regarding NF mRNAs, the conserved 3′UTR seems to serve as a hub for modifications, as many RBPs (TDP43 (Strong et al., 2007), 14-3–3 (Miao et al., 2013), Aldolase A/C (Cañete-Soler et al., 2005), FUS (Lagier-Tourenne et al., 2012) bind to this region to either stabilise, destabilise or modulate the mRNA of NF-L, NF-M and NF-H. MiRNAs (miR-b1336 (Ishtiaq et al., 2014), MiR-146a (Campos-Melo et al., 2013), miR-233-3p (Campos-Melo et al., 2018), whose biogenesis can be controlled by RBPs, also bind to the 3′UTR regions of the NF mRNAs. Other RBPs (hnRNP K (Thyagarajan and Szaro, 2004; 2008), ELAVL2 (Antic et al., 1999)) are capable of modulating mRNA transport and translation, hence directing the local synthesis of NF proteins in neuronal sub-compartments. Less is known for α-internexin and peripherin, with the RBP hnRNPK modulating only α-internexin mRNA translation. Overall, studies on post-transcriptional modifications revealed that protein synthesis does not occur exclusively in the soma, but that NF mRNAs can be protected/destabilised along their journey, delivered and translated in different compartments, therefore providing the neuron with a high level of dynamics in fulfilling local demands and responding to stimuli.

**FIGURE 3:**
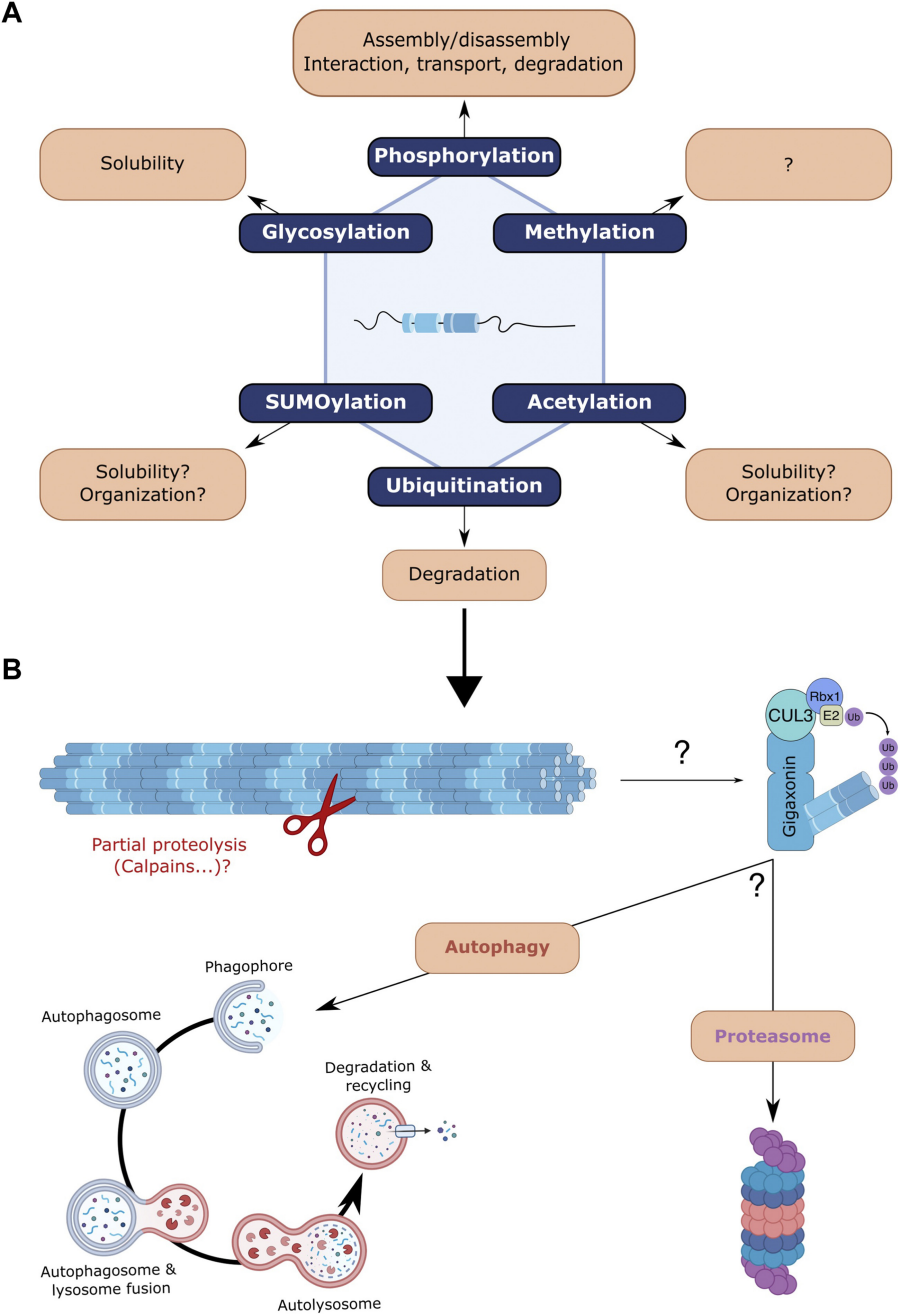
Post-translational modifications of neurofilaments and focus on ubiquitin-dependent degradation. **(A)** NFs undergo various post-translational modifications (PMTs) that can regulate their behaviour: methylation, acetylation, SUMOylation, glycosylation, phosphorylation and ubiquitination. Very little is known regarding the effects of most NF-specific PTMs but findings from other IFs may suggest a role in regulating filament solubility and organisation (acetylation, SUMOylation). Glycosylation was also shown to modulate the solubility of NFs. Phosphorylation, the most studied PTM has revealed various roles, including filament assembly/disassembly, interaction with NFs and other cytoskeletal components, NF transport and protection from proteolytic degradation. **(B)** Focus on the ubiquitination and proteolysis of NFs. While the Trim2-E3 ligase was shown to ubiquitinate NF-L, gigaxonin (which at least induces peripherin ubiquitination) is the only E3 ligase capable of degrading all NF subunits. Whether the gigaxonin-mediated clearance is dependent on the proteasome and/or autophagy pathways remains to be determined, but autophagy and proteasome have been identified as degradative routes for NF proteins *in vivo*. Various unsolved questions concern the early requirement of partial proteolysis by proteases, the NF forms that are substrates for proteolysis, the cooperation of both degradative routes and the spatio-temporal signalling that activates and regulates them.

Post-translational modifications (PTM) of proteins play an important role in physiology by modulating various properties including protein activity, interaction, subcellular localisation, stability and turn-over. Proteomic analyses have identified or predicted specific sites on NF proteins that can be modified by PTMs (MacTaggart and Kashina, 2021). NFs can be the targets of various PTMs, including glycosylation, acetylation, SUMOylation, methylation, but the most studied are phosphorylation and to a lesser extent ubiquitination (Figure 3).

The three polypeptides (NF-L, NF-M and NF-H) were the first IFs identified as being subjected to phosphorylation (Pant et al., 1978) (Figure 3A). The level of phosphorylation of NF-M and NF-H is found to be particularly high when compared to the basal phosphorylation level of other IFs. This is related to their particularly long tail domains rich in lysine-serine-proline (KSP) motifs. All NF subunits can be phosphorylated in their head domain, but only NF-M and NF-H are highly phosphorylated in their tail domains (Dale and Garcia, 2012; Holmgren et al., 2012). Interestingly, this phosphorylation in different portions of NF proteins is modulated in distinct subcellular compartments. Thus, the head domain is more phosphorylated in the soma, while the tail domain is more phosphorylated in the axons (Sihag et al., 2007). This implies a tight spatio-temporal regulation of kinase/phosphatase during development, when newly synthetised NF proteins in the cell body enter the axons. Phosphorylation in the head of NF subunits (mediated by PKA, PKC and CAMK II, cdk5 … ) has been mainly shown to regulate the assembly-disassembly of the filament, but it is also exerting negative control of tail domain phosphorylation, presumably to avoid abnormal stabilisation and aggregation of NFs in the soma (Yuan et al., 2017). NF tail domain phosphorylation (mediated by CDKs, MAPKs … ) occurs mostly at the KSP sites and preferentially upon their entry into the axons. Interestingly, the degree of phosphorylation increases as the NFs progress distally into the axons, therefore contributing to the maturation of the filament and the interaction with other cytoskeleton components. Accordingly, myelination and synaptogenesis regulate the phosphorylation of NF tail domains, inducing NF spacing and promoting axonal radial outgrowth (Lariviere and Julien, 2004; Holmgren et al., 2012).

Collectively, NF phosphorylation plays a crucial role in the formation, maintenance and remodelling of the NF network (this will be expanded in the following sections), and alterations of this balance are associated with the settings of neurodegenerative diseases (Dale and Garcia, 2012; Binukumar et al., 2013).

Ubiquitination is a PTM involved in the turn-over of NF proteins (Figure 3B). With an estimated half-life of >8 months in sciatic nerves, the NF array was initially shown to be extremely stable *in vivo*. Surprisingly, genetic alterations of NFs evidenced a certain dynamic in the NF array. Thus, NFs were found to be degraded in a synchronous manner along the nerve and much faster (estimated 3 weeks half-life) in the absence of a preexisting NF array (Millecamps et al., 2007). Interestingly, the tail domains of the NF-M and NF-H proteins are critical as they protect the filament *in vivo* from proteolytic degradation (Rao et al., 2012). Also, NFs, when incorporated in the array, exhibit a slower decay than the surrounding less-phosphorylated NF particles (Boumil et al., 2018). Therefore, the NF array itself, whose density varies between neuronal subtypes and along individual axons, is a key determinant of NF stability within neurons. The mechanisms leading to the degradation of the NF array are not fully elucidated but the gigaxonin-E3 ubiquitin ligase (Bomont et al., 2000) has been found to be a universal regulator of IF steady state (Bomont, 2016). While the absence of gigaxonin in patients suffering from a neurodegenerative disease called GAN induces an abnormal accumulation of several IF types (Lescouzères and Bomont, 2020), its overexpression has been shown to induce the clearance of several IF types, including the NF-L, NF-M, NF-M, peripherin and α-internexin (Israeli et al., 2016). Extremely difficult to evidence, ubiquitination has so far only been revealed for peripherin (Israeli et al., 2016). Another E3 ligase (Trim2) has been shown to ubiquitinate NF-L (Balastik et al., 2008), although it does not regulate NF abundance (Li et al., 2020). The generalised action of the gigaxonin relies on its interaction with the central rod domain, which is conserved between all IF types (Mahammad et al., 2013). Many areas require further investigation, such as the forms that are degraded (filament, ULF, tetramers), the prerequisite action of calpain, the calcium-activated protease necessary for NF degradation in Wallerian degeneration (Glass et al., 1994; Park et al., 2013a; 2013b; Ma et al., 2013). Regarding NF proteins, very little is known about the implication of the proteasome but recent evidence points towards the autophagy pathway. Firstly, the gigaxonin-E3 ligase was shown to regulate the flux of the autophagy pathway (Scrivo et al., 2019), by promoting the elongation of the autophagosomes (Bomont, 2019; Lescouzères and Bomont, 2020). Secondly, the abundance of the NF triplet (NF-L, NF-M and NF-H) has been shown to be regulated by autophagy activity *in vitro*, to a greater extent than the proteasome (Rao et al., 2023). In mouse brains, the levels of NF-M and NF-H (but not NF-L) could be increased by < 2 fold upon inhibition of the autophagic flux, while proteasome inhibition has less effect and only on NF-M. Furthermore, NF particles have been detected in the lumen of double-membrane autophagosomes but with no filamentous structure, suggesting that shorter processed NFs (oligomers, ULF, monomers … ) are addressed to the autophagic pathway (Rao et al., 2023). Altogether, these findings reveal that NF proteins are substrates for both the proteasome and the autophagy pathways, with a specificity towards subunits that remains to be scrutinised. In the future, it would be important to determine the spatio-temporal variations of intrinsic (structure, PTM of subunit, assembly state … ) and extrinsic (composition of degradative machineries, cooperation between systems, inducers … ) properties of these crucial regulations sustaining NF steady state.

### 2.4 The dynamics of neurofilaments

In contrast to the microtubule and actin networks, which are remodelled by rapid depolymerisation and polymerisation waves, NFs have long been considered as an extremely stable and non-dynamic network. Still, NF particles were found to incorporate and leave the NF array (Nixon and Logvinenko, 1986; Lewis and Nixon, 1988). The apolar structure of NFs represents a major hurdle to investigate the dynamic aspects of the network, but the development of methodologies adapted to IFs has allowed us to examine the behaviour of NFs.

**FIGURE 4:**
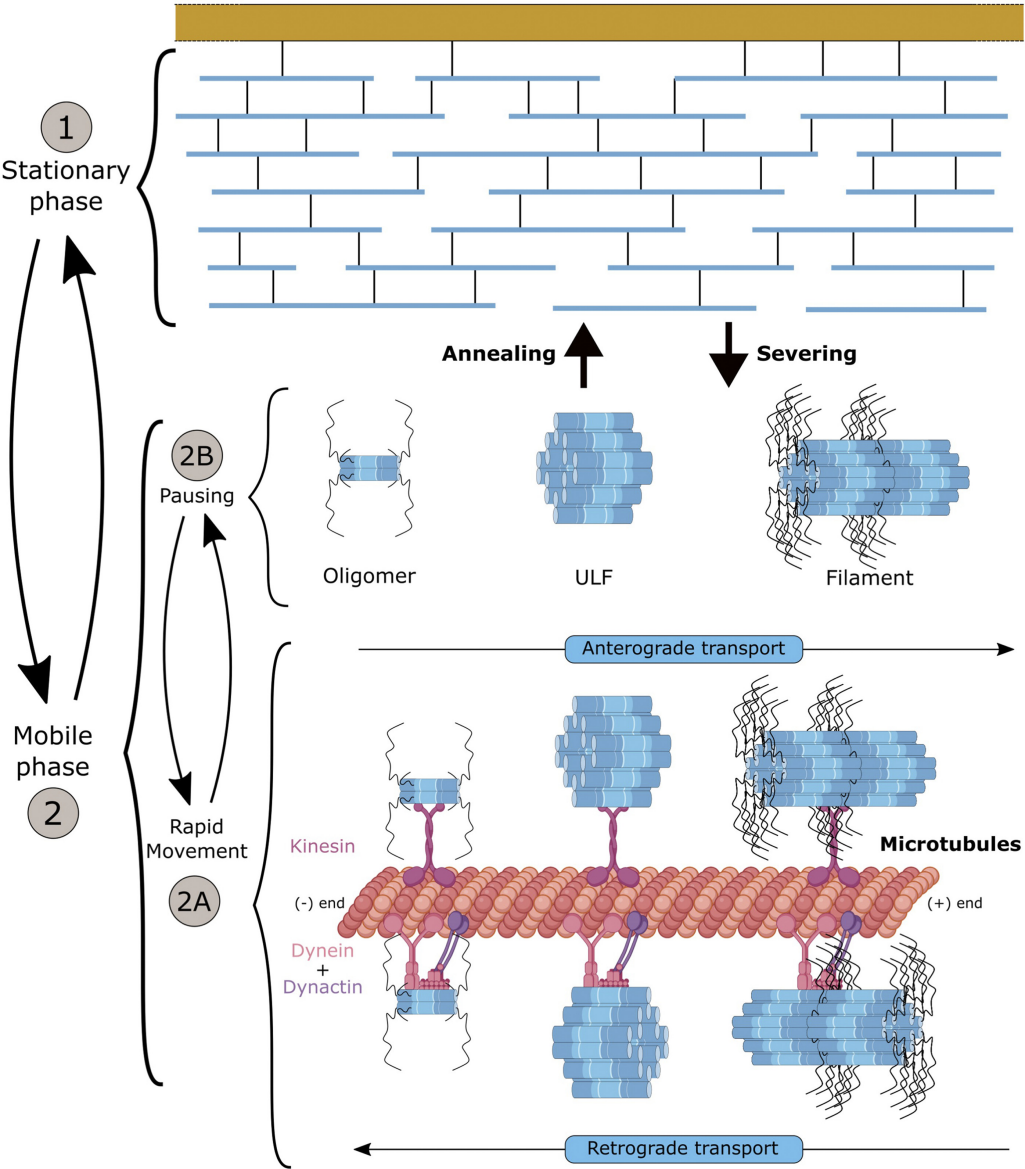
Axonal transport of neurofilaments and formation of the network. NFs can be distinguished into two phases: (1) the stationary phase and (2) the mobile phase into which NFs can enter cyclically. The mobile phase appears as a “slow” transport but results from bidirectional and net “fast” movements powered by the dynein and kinesin motors onto microtubules (2A), interrupted by short pauses (2B). Distinct NF structures (heterooligomers, ULF or short filament) that coexist in the same axon can be further incorporated into the stationary network for prolonged pausing. Exchange of NF portions has been shown to occur along the filament through annealing and severing. Overall, NFs spend 97% of their time in an immobile phase (short and long pauses), but it is assumed that NFs can cycle between the different phases, to provide to the abundant NF network the dynamics necessary to adjust NF content and respond to developmental stimuli and insults. The mechanisms controlling the switch from movement to pausing (2A-2B) and integration into the filament (1) are not fully understood, but they may involve many factors (detachment of NFs from molecular motors, access to MTs, etc.). Likewise, the structure of the NFs, and the regulatory elements enabling local annealing and severing without compromising the entire filament are not known.

As discussed earlier, NF subunits exhibit a sequential expression pattern accompanying key stages of neuronal maturation (Figure 2). Synthetised in the cell body, NF proteins are then transported to the axonal compartment (Yuan et al., 2009). Over the last decades, two critical aspects of NF maturation have been the subject of intense debate, with conflicting findings obtained as a result of the different model systems and methodologies used to visualise the NF array: the identity of the NF structures transported along the axon and the characteristics of transport (Figure 4). In both cases, the emergence of optical methodologies adapted to IFs and the manipulation of individual subunits permitted to evidence that multiple assembly forms of NFs are transported along the axon, and that the apparent slow axonal transport of NFs is driven by a net fast movement but with intermittent prolonged pausing.

It is worth noting that the outcomes obtained in studies performed *in vitro* and *in vivo* should take into account the great variability of NF content between both systems and may not be comparable. Thus, adult mice axons express NF levels that can exceed 100–1,000 folds of what is present in embryonic primary neurons.

Regarding the first aspect, NFs were shown to be transported either in the form of non-filamentous NF structures, oligomers or short polymers (Yuan et al., 2017). Interestingly, these forms can co-exist in the same axon, with a spatial localisation that would suggest an assembly into filament as the NF structures are transported along the axon (Yuan et al., 2009). Additional findings *in vivo* showed that the formation of a mature NF network is not a prerequisite for the transports of NF forms (Millecamps et al., 2007), and that NF content and variation along the axon does not regulate the rate of NF transport (Rao et al., 2012; Yuan et al., 2015a), even-though NF removal from axons induces a faster NF transport rate (Millecamps et al., 2007). The life cycle of NFs was originally proposed to begin by expression of NF subunits in the soma, followed by the transport of NF structures along the axon and network formation, and finally the degradation of the entire pool of NFs as they progress through the synaptic terminals. It is now shown that as in degradation, NF transport can occur all along the axon, but only a small population of NF structures is transported at any given time, while the majority of the array is stationary (Figure 4). This would enable the neuron to maintain an abundant NF network at a minimal energy cost along the entire length of the long axon, while locally adjusting NF content in response to myelination and various environmental signals.

Initial studies performed *in vivo* using radioisotope pulse labelling established that NFs move in the slow compartment (0.25 mm/day) (Black and Lasek, 1980), but in two distinct pools with one being in continuous motion and the second being more stationary (Nixon and Logvinenko, 1986). *In vitro* studies in neuron types presenting a scarcity of NFs, combined with the development of live-cell imaging (photo-bleaching/activating/conversion) and computational methodologies enabled to scrutinise the characteristics of NF transport. Collectively, in cellular system, the global slow transport of NFs was shown to result of a net fast transport (50–400 mm/day) that is interrupted by prolonged pauses (Roy et al., 2000; Wang et al., 2000). With an average velocity of 1  ym s^-1^ (Fenn et al., 2018), NF transport is bidirectional and powered by molecular motors. More specifically, interactions of specific NF subunits with MT-based and actin filament-based motors were identified: KIF5A with NF-M and NF-H (Yabe et al., 2000; Jung et al., 2005), dynein with NF-M (Wagner et al., 2004) and myosinVa with NF-L (Rao et al., 2002a). Consistent with the abnormal accumulation of NFs in the cell body in KIF5A knockout mice (Xia et al., 2003) and altered NF transport in KIF5A KO neurons (Uchida et al., 2009), overexpression of KIF5A mutants (as identified in the SPG10 disease, a form of human hereditary spastic paraplegia) evidenced a defect in the frequency of NF transport along the axon (Wang and Brown, 2010). Disrupting dynein was shown to reduce the (retrograde) transport of NFs (He et al., 2005; Uchida et al., 2009). Myosin-depleted neurons revealed a prolonged stay of NFs in the stationary network, suggesting a role in engaging NFs onto the MT network (Alami et al., 2009). While interactions with the molecular motors and their cooperativity in promoting the bidirectional motility of different forms of NF structures are not fully characterised, a general model for NF transport has been proposed. In the current model (Figure 4), the mobile phase is characterised by the bidirectional transport of various NF forms along MTs, with a fast net transport powered by molecular motors that is intermittent and interrupted by short pauses (estimated at 30 s). NFs can switch to a stationary phase, in which NFs undergo prolonged pauses (estimated at 60 min), presumably as a result of their incorporation into the stable interconnected filament. Overall, NFs pause 97% of the time, which would explain their global slow transport of 0.5 mm/day (Trivedi et al., 2007). While many aspects of this model remain to be investigated, the model puts forward the argument that the short pausing behaviour may be due to the detachment of molecular motors from NFs, and that the incorporation into the stationary network may result from the unavailability or prevention of reattachment to motors or weak MT density (Shea, 2000). This model has been essentially built in cellular systems conveniently presenting a scarcity of NFs. Therefore, it may present different characteristic *in vivo*, in nerves presenting significantly higher NF density.

The second subject of debate concerns the capacity of all NFs, including those present in the stationary network, to switch to a mobile state. The first view, based on computational analysis, proposes a single NF population model in which transported NFs form the entire NF content of the axon (Brown and Jung, 2013), that would suggest a high dynamic of the network and imply weak and reversible interactions between filaments. Accordingly, *in vivo* assessment of NF motility in large myelinated axons revealed a bidirectional spread from the photoactivated area (Boyer et al., 2022) that involved >80% of the network, following 3 h of activation (Fenn et al., 2023). The second view, supported by various anterior *in vivo* studies, proposes a two-NF population model in which transported NFs represent a small fraction maintaining a long-lived stationary network (Yuan et al., 2015a). In this situation, the local variation of NF content along the axon would not be determined by NF transport, but instead by the rate of incorporation of NFs into the filament, and by the half-life of the stationary NFs. The presence of the stationary NF network would permit the neuron to maintain axonal integrity at low energy cost while permitting local NF density regulation in response to various stimuli. Solving the issue of the population undergoing motility in axons is important but complex, and discrepancies cannot simply be attributed to the biological system, as respective studies have been performed *in vivo*.

Regarding the possible mode of integration of moving NF particles into the preexisting filaments, *in vitro* studies revealed that subunit exchange and severing occur along the length of the filament (Colakoğlu and Brown, 2009; Uchida et al., 2013). Presumably, the filament would have a topologically open architecture enabling portions of the filament to dissociate or associate without compromising the integrity of the network. While little is known regarding the pathways that control NF behaviour, myelination (Monsma et al., 2014, 201; Walker et al., 2019) and phosphorylation of the NF-H (or NF-M) tail domain (Jung et al., 2000a; 2000b; Ackerley et al., 2003) that are argued to detach NFs from motors (Shea and Flanagan, 2001; Holmgren et al., 2013) have been identified as negative regulators of NF motility. Surprisingly, depletion of the heavily phosphorylated tail of either NF-H (Rao et al., 2002a; Yuan et al., 2006), NF-M (Rao et al., 2003) or both (Rao et al., 2012) does not alter NF transport in optic nerve axons. While this does not exclude a pathological role attributed to tail phosphorylation in modulating NF transport (i.e., hyperphosphorylation), these findings may suggest relevant phosphorylation sites outside long tail domains, as suggested by the partial NF transport defect upon phosphorylation manipulation in the NF-L head domain (Yates et al., 2009). Equally puzzling is the finding that the gigaxonin-E3 ligase, responsible for the degradation of IFs was recently shown to modulate NF transport in dorsal root ganglion (DRG) sensory neurons (Renganathan et al., 2023).

### 2.5 Roles of neurofilaments in neuronal physiology

**FIGURE 5:**
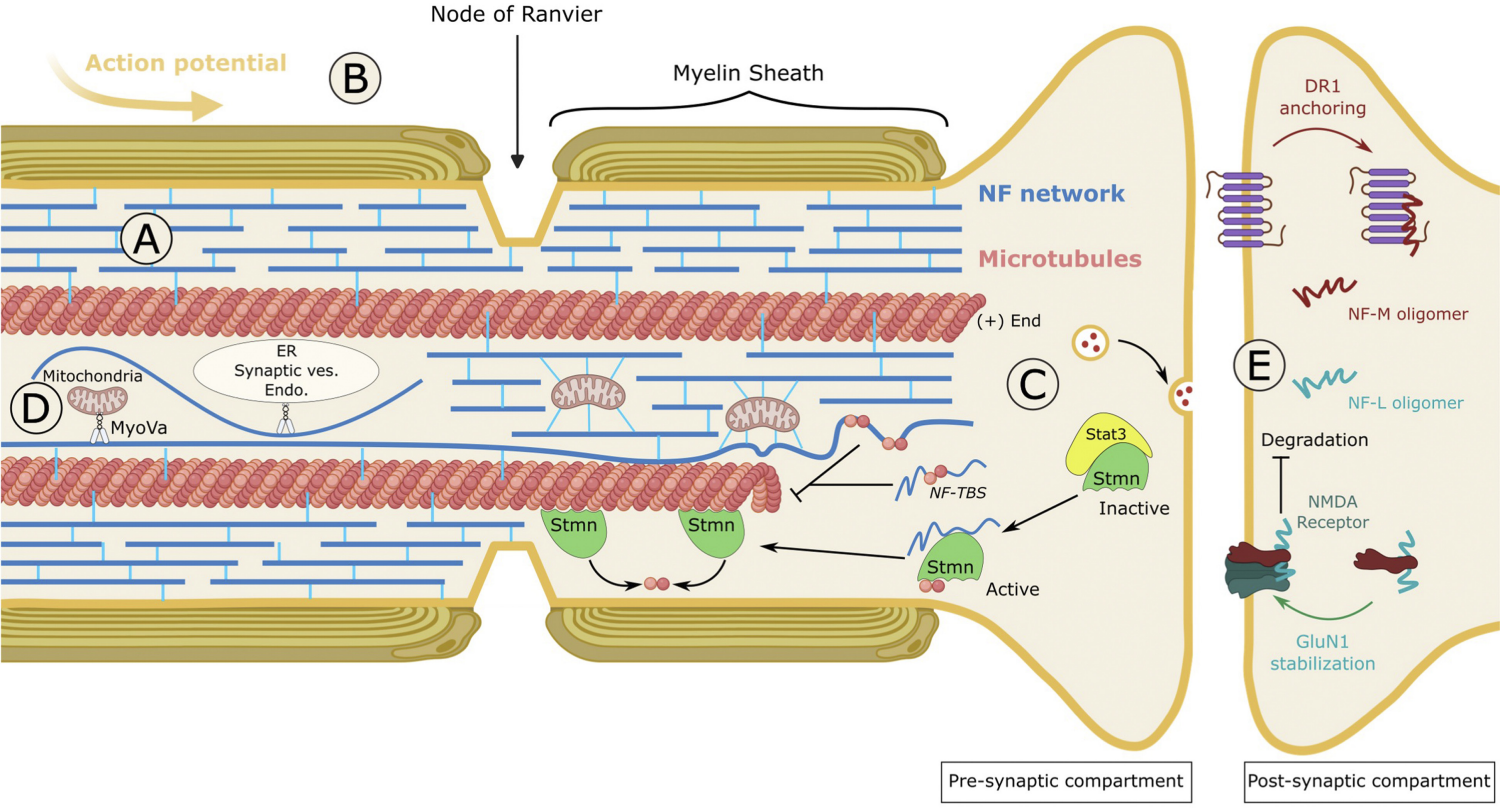
Cellular and physiological roles of neurofilaments. **(A)** Structural support of the axon. NFs form an abundant interconnected network that exhibits exceptional viscoelastic properties. Resisting an average stretching of 260%, NFs confer a mechano-resistance to long axons. **(B)** Axon diameter and nerve conduction. NF density and spacing within the axon are key determinants of radial axonal outgrowth, myelination and conductivity. Large, myelinated axons are filled with interspaced NFs. Many conditions decreasing the axonal content of NFs or the protrusion of their tails alter axonal caliber and nerve conduction. One puzzling finding is the greater role of the NF-M tail, which is shorter than the NF-H tail. **(C)** Regulator of microtubule dynamics. NFs act as a negative regulator of MT dynamics through two mechanisms. On the one hand, a tubulin-binding site (TBS), present in the head domain of NF proteins binds to and sequesters unassembled tubulin, therefore inhibiting MT polymerisation. On the other hand, NFs disrupt the inactive Stat3-Stmn complex and release Stmn which is a microtubule-destabilising factor. In conditions where MTs are destabilised, NF depletion can promote the formation of Stat3-Stmn complex, hence stimulating the regrowth of MTs. **(D)** Distribution of organelles. Most abundant scaffold of the cell, NFs play a role in the positioning of organelles. The direct interaction of NFs with mitochondria anchors them to the network, hence decreasing their motility on MTs. Alternatively, the binding of NFs to the actin-molecular motor Myosin Va (MyoVa) regulates the distribution of the ER, mitochondria and endosomes within the axon, possibly by permitting exchange between actin, NFs and MTs. **(E)** Neurotransmission. Exhibiting different assemblies than those found in the axon (short oligomers), synaptic resident NFs regulate receptor trafficking and stability at the membrane of the post-synaptic compartment. NF-M associates with the D1R to anchor the internalised receptors on endosomes, to promote desensitisation to D1R stimulants such as cocaine. NF-L associates with GluN1, one of the subunits of the NMDA receptor, and stabilises it at the plasma membrane by protecting it from ubiquitin-dependent degradation. Thus, NF-L regulates the density and length of dendritic spines and stimulates various NMDAR-dependent responses such as spatial and social memory. NMDAR: N-methyl-D-aspartate receptor.

Gene manipulations in mice, along with the identification of causal genes in neurodegenerative diseases, demonstrated the importance of NFs in neuronal physiology and shed light into to the molecular mechanisms sustaining various functions (Figure 5) (Yuan et al., 2017; Bomont, 2021).

As the most abundant constituent of mature neurons, NFs have long been known for the protection they provide to long axons from mechanical insults (Figure 5A). Intensively inter-connected through the tail domains of NF-M/H, the NF array presents structural properties that can resist an average stretching of 260% before rupture (Kreplak et al., 2005; Sapra and Medalia, 2021). The exceptional viscoelasticity of this scaffold provides at the same time the mechano-resistance necessary to protect axons during the lifetime of an individual and adaptation to environmental stress.

Many studies have established that NFs are essential for the radial growth of large myelinated axons and for nerve conduction (Figure 5B). NF content has been shown to correlate with axonal diameter (and is reduced at the node of Ranvier), and the protrusion of NF tails outwards the highly dense filament was evidenced as a key determinant of NF spacing and radial axonal outgrowth (Lariviere and Julien, 2004). Thus, genetic manipulations to decrease NF density or spacing result in the reduction of axon caliber and nerve conduction. Amongst NFs, NF-L was shown to be a major determinant of NF content in axons. Indeed, depletion of NF-L through genetic manipulation (Zhu et al., 1997), in a spontaneous quail line (Sakaguchi et al., 1993) and in CMT patients with recessive mutations in the *NEFL* gene (Sainio et al., 2021) substantially inhibits radial growth and nerve conduction. Interfering with NF spacing has a similar effect on decreasing axonal caliber and conductivity but, surprisingly, the NF-M tail domain (as seen in the NFM-tailless (Rao et al., 2003), NFM/H tailless mice (Garcia et al., 2003)) and not the NF-H tail was shown to be the key determinant (Rao et al., 2002b). While this would suggest that the specific phosphorylation of KSP repeats within NF-M is the driver of radial axonal outgrowth, the systematic inactivation of all known KSP sites in the NF-M tail does not alter axonal caliber (Garcia et al., 2009). Another unexpected finding is the regulation of internode length (the distance between the nodes of Ranvier) by the phosphorylation of the tail domain of NF-M (Villalón et al., 2018).

Interconnected with the actin filaments and microtubules, NFs have been shown to regulate MT dynamics (Figure 5C). This line of research emerged from the observation (carried out in NF content-lowering conditions as seen in NF mouse models and the quail line) that MT and NF densities are inversely correlated, sometimes without changing the level of soluble tubulin (Eyer and Peterson, 1994; Zhao and Szaro, 1995; Zhu et al., 1997; Elder et al., 1998; Jacomy et al., 1999; Bocquet et al., 2009). Locally, MTs have also been shown to be reduced or excluded from densely packed NFs in various diseases, such as GAN (Donaghy et al., 1988; Ganay et al., 2011), suggesting a decreased ability of MTs to polymerise in NF rich regions. In NFH^LacZ^ transgenic mice, NFs aggregate in the soma, preventing their export to the axon which are therefore depleted in NFs. Examination of the distribution of MTs and tubulin levels revealed that while axons lacking NFs are filled with MTs, the soma packed with NFs are devoid of MTs, even with normal tubulin levels (Bocquet et al., 2009). The negative effect that NFs exert on MT dynamics is mediated by two mechanisms. On one hand, a tubulin-binding site (TBS) identified in the head domain of NF proteins has been shown to bind soluble tubulin and to be sufficient to inhibit MT polymerisation (Bocquet et al., 2009). Therefore, NFs would act as a reservoir for unassembled tubulin, providing a source throughout the axon for rapid local polymerisation. On the other hand, NFs have been shown to destabilise MTs through disruption of the stathmin complex. This has been revealed in mice with progressive motor neuronopathy (pmn), characterised by a drastic destabilisation of MTs due to *TBCE* mutations and an increase in NF content. Depletion of NFs in the pmn model results in the regrowth of MTs, due to the inactivation of the MT-destabiliser stathmin, through increased interaction with Stat3 (Yadav et al., 2016).

Considering the key role of MTs for axonal transport of organelles, the regulatory action that NFs exert on MTs dynamics is likely to impact the distribution of organelles. More directly, NFs also constitute a cellular scaffold for the docking and organisation of organelles (Figure 5D). NFs have been shown to interact with organelles either directly (i.e., phosphorylated NFs binds to mitochondria (Wagner et al., 2003)), or through the actin-molecular motor MyosinVa (MyoVa) (Rao et al., 2002a). Thus, the motility of mitochondria is regulated by NF density. It is increased in the absence of NFs as seen in the NF-L KO mice and recessive NF-L CMT (Perrot and Julien, 2009; Gentil et al., 2012; Sainio et al., 2021) and decreased upon NF aggregation as seen in dominant NF-L CMT and GAN (Gentil et al., 2012; Saporta et al., 2015; Israeli et al., 2016; Nath et al., 2023). While these data suggest that NFs serve as a docking site, other NF-independent mechanisms susceptible of redistributing mitochondria to cope with insult may be considered (Van Lent et al., 2021). Surprisingly, NF-L interacts with the motor domain of MyoVa (Rao et al., 2002a; 2011), an actin-based molecular motor which transports many cargoes, including synaptic vesicles, endosomes, mitochondria, and endoplasmic reticulum (ER). NF-L depletion was shown to induce a redistribution of the ER, mitochondria, and endosomes to the periphery, hence revealing that NFs are crucial for MyoVa-mediated distribution of organelles within the axon (Rao et al., 2011). It is hypothesised that by binding to the motor domain of MyoVa, NFs may anchor organelles along the axon and facilitate their distribution across actin and MTs that are highly interconnected.

NFs are also critical regulators of neurotransmission (Figure 5E). Synapses have long been considered as the degradative site for NFs and, as a result, NF presence in this compartment was considered as an axonal contaminant (Yuan et al., 2017). More recent data has proven this to be wrong, with synaptic NF assemblies being very specific and playing crucial roles in synapse functions. NFs isolated from synapses present differences from axonal NFs, with a higher proportion of α-internexin and reduced phosphorylation of NF-M (Yuan et al., 2015b). This distinct population of NFs is more abundant in the post-synaptic compartment and can only occasionally form 10 nm short polymers. These characteristics may suggest an immature-like state of NFs (such as during development) that would confer greater local plasticity to the cytoskeleton. Considering that monomers are inefficiently transported along the axons, synaptic NFs would be at the very least transported in an oligomeric assembly form or be alternatively synthetised from mRNA at the synapse (Yuan et al., 2015b). Gene depletion studies in mice revealed the crucial roles of NFs in regulating receptor targeting and stability at the post-synaptic membrane, as well as substantial alterations in neurotransmission and behaviour.

NF-M, which was previously shown to interact with the dopamine D1 receptor (D1R) (Kim et al., 2002), plays a role in its recycling to the membrane by anchoring internalised D1R on endosomes at the synapse lumen (Yuan et al., 2015b). Thus, NF-M deletion increases the redistribution of post-synaptic D1R from endosomes to the membrane and amplifies dopamine-D1R-mediated motor responses to cocaine. NF-L was reported to interact with the glutamatergic GluN1, one of the four subunits of the N-methyl-D-aspartic acid receptor (NMDAR) (Ehlers et al., 1998). A study revealed that NF-L stabilises GluN1 at the membrane, by protecting it from ubiquitin-dependent degradation. In agreement with the known role of the NMDAR in regulating dendritic spine morphology, NF-L depletion, which decreases GluN1 level *in vivo*, also decreases the density and length of dendritic spines (Yuan et al., 2018). Moreover, NF-L depletion was shown to alter neurotransmission and cause various behavioural defects as seen in NMDAR hypofunction, included but not restricted to spatial and social memory. Altogether, these findings show that a unique population of NFs in the synapses plays key roles in neurotransmission *in vivo* and may suggest a potential implication in addiction, memory, and psychiatric and neurological diseases (Yuan and Nixon, 2016).

## 3 Neurofilaments: pathological cause of Charcot-Marie-Tooth disease

Neurofilaments sustain critical functions from neurodevelopment to adulthood, and alterations of this network are the direct or contributing causes of various neurodegenerative diseases in humans (Didonna and Opal, 2019; Bomont, 2021). Thus, abnormal aggregation of NFs represents one of the earliest pre-symptomatic hallmarks for most, if not all, neurodegenerative diseases. Interestingly, rare NF variants (possibly polymorphisms or gene modifiers) have been associated with amyotrophic lateral sclerosis, Alzheimer’s and Parkinson’s disease (Yuan et al., 2017), but their role in pathogenicity is still uncertain. In addition, several NF-controlling genes have been identified as the genetic cause of numerous neurological diseases. These include NF assembly (Sacsin, HSPB1), NF transport (KIF5A) and NF degradation regulators (gigaxonin) (Bomont, 2021). In these pathologies, determining the contribution of NFs to disease is challenged by the fact that the respective proteins regulate multiples targets and functions. In this review, we will focus on Charcot-Marie-Tooth (CMT) disease, for which *NEFL* and *NEFH* are causal genes. It is still important to highlight that NF mutations can also cause hereditary spastic paraplegia (*NEFL*), spinal muscular atrophy (*NEFH*) but also myopathy (*NEFL*), which evidence a broader phenotypic spectrum of NF mutations (Wang and Brown, 2010; Agrawal et al., 2014; Ando et al., 2022).

### 3.1 Charcot-Marie-Tooth: a heterogeneous group of diseases

Charcot-Marie-Tooth disease, also known as hereditary motor and sensory neuropathy (HMSN) is the most common inherited neurological disorder of the peripheral nervous system, with a worldwide prevalence rate of 1 in 2,500 (Laurá et al., 2019; Rudnik-Schöneborn et al., 2020). CMT is a motor and sensory neuropathy caused by the progressive deterioration of the peripheral nerves, composed of the lower motor axons projecting from the spinal cord and the sensory axons from the dorsal root ganglia. Thus, CMT is most commonly characterised by muscle weakness, amyotrophy and sensory loss that start in the lower limbs and progress slowly in a length-dependent manner. A high incidence of foot deformities (*pes cavus*) is also seen in CMT. There is considerable variability concerning the age of onset, the severity of the symptoms and the progression of the disease between different forms of CMT, even in the same family. Most patients present symptoms in the first or second decade of life, although it may also start in infancy or at an advanced age. The clinical presentation of the disease can be highly variable. Indeed, more severely affected patients may become non-ambulant by the third decade of life, whereas other individuals can only present minimal symptoms such as *pes cavus* or minor distal muscle atrophy. Some mutation carriers may even age without experiencing any clinical signs. Historically, CMT was described as a group of disorders primarily affecting the peripheral nervous system, but in recent years many forms of other non-neurological and neurological symptoms within the central nervous system have been found (Pipis et al., 2019), including dementia, cerebellar syndrome, hearing impairment and optic atrophy.

CMT is classified into two main types, according to neurophysiology and histopathological features at the nerve biopsy: the demyelinating (CMT1) and the axonal (CMT2) forms (Laurá et al., 2019; Rudnik-Schöneborn et al., 2020). CMT1 is characterised by a reduction of the upper limb motor nerve conduction velocities (MNCV) (below 38 m/s) and a predominant alteration of the Schwann cells, the glial cells of the peripheral nervous system that form the myelin sheath around the nerve fibers necessary for saltatory conduction. CMT2 has a MNCV above 38 m/s and is primarily caused by axonal degeneration. Another form, the intermediate form (I-CMT), has emerged, with MNCV ranging between 25 m/s and 45 m/s (Berciano et al., 2017). Considering the invasiveness of sural nerve biopsy, the associated pain and poor diagnostic value in pinpointing specific genes, this procedure is no longer conducted in regular practice. Confusingly, recessive forms of CMT1 have been named CMT4. CMT are further classified according to the mode of inheritance (mostly dominant but also recessive and X-linked) and the genetic cause. The most common form of CMT (60% of cases) is CMT1A, which is caused by a genomic duplication comprising the peripheral myelin protein 22 gene (*PMP22*). In CMT2 (which represents about 30% of all CMTs), the mitofusin gene (*MFN2*) is the most common form (CMT2A), which accounts for 20% of axonal CMT. The development of next-generation sequencing (NGS) technologies (gene panels, whole-exome/whole-genome/mitochondrial/high-throughput transcriptome sequencing) revolutionised genetic diagnosis and allowed the number of genes to be considerably expanded to >100 for CMT (Rossor et al., 2013; Pipis et al., 2019). This revealed the high genetic heterogeneity, which is further complicated by the fact that several genes can cause different forms of CMT. For example, the *NEFL* gene, originally discovered in a Russian family suffering from axonal CMT2E, has been further implicated in CMT1F and I-CMT.

### 3.2 The NF-Charcot-Marie-Tooth

**FIGURE 6:**
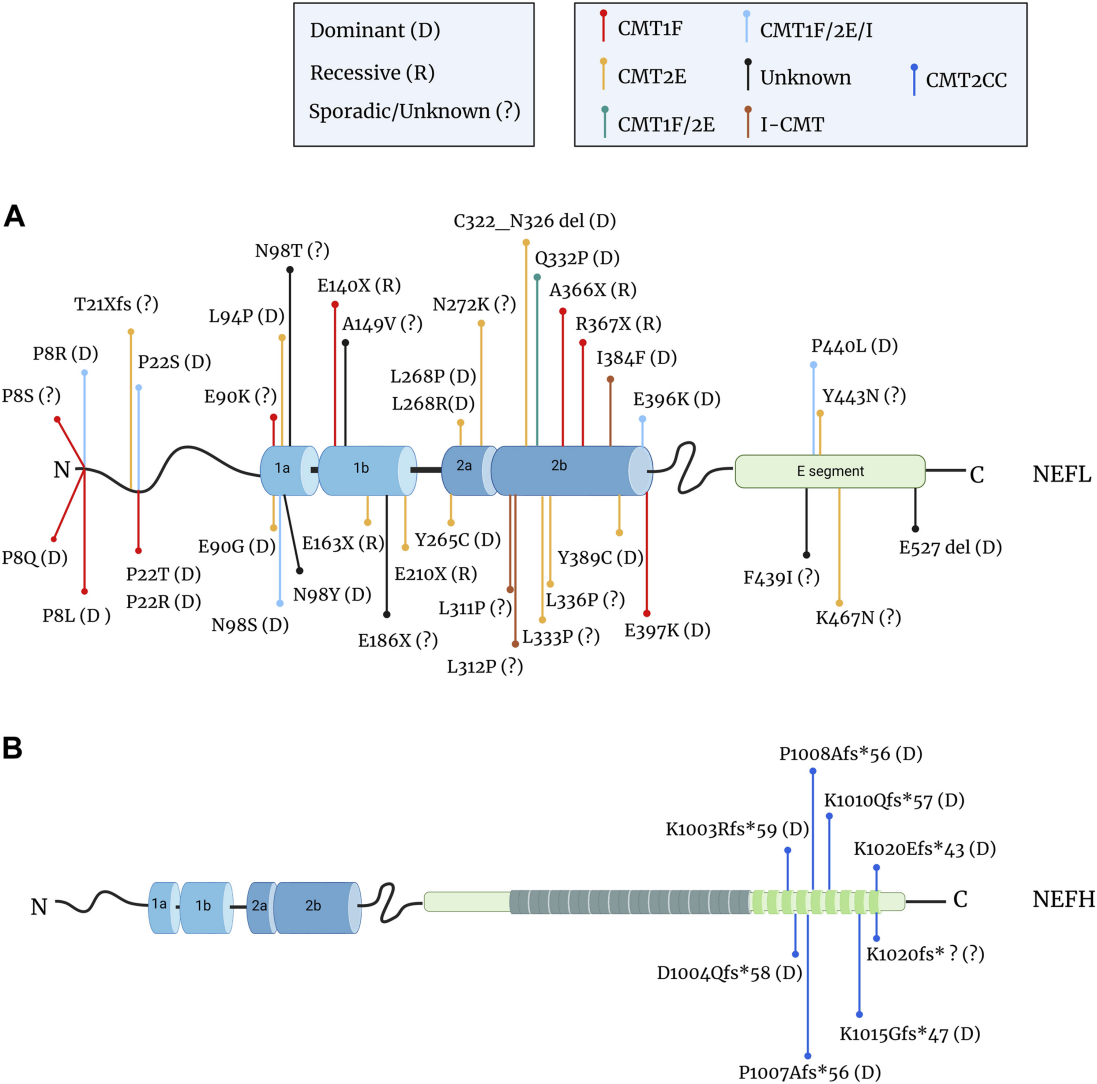
Mutations in the *NEFL* and *NEFH* genes in CMT patients. **(A)** Localisation of the mutations in the NF-L protein that cause demyelinating CMT1F (red dot), axonal CMT2E (yellow dot), intermediate I-CMT (brown dot), unknown CMT (black dot) or mixed forms: CMT1F or CMT2E (green dot); CMT1F, CMT2E, or I-CMT (light blue dot). Missense mutations are the most common, but frameshift (fs), deletion (del), and nonsense mutations (X) have been reported. Mutations are often inherited through a dominant mode (D) but recessive transmission (R) also occurs and several cases are undetermined or sporadic (?). The nonsense mutations are located within the rod domain and are all recessive, while missense and in-frame deletion mutations are dominantly inherited. **(B)** Mutations in the tail domain of the NF-H protein cause axonal CMT2CC (dark blue dot). Nucleotide changes (e.g., deletion, insertion) create frameshift mutations that are inherited through an dominant mode or *de novo*. Data from: (Mersiyanova et al., 2000; De Jonghe et al., 2001; Georgiou et al., 2002; Yoshihara et al., 2002; Jordanova et al., 2003; Choi et al., 2004; 2012; Fabrizi et al., 2004; 2007; Züchner et al., 2004; Leung et al., 2006; Miltenberger-Miltenyi et al., 2007; Shin et al., 2008; Abe et al., 2009; Bhagavati et al., 2009; Yum et al., 2009; Benedetti et al., 2010; Lin et al., 2011; Sivera et al., 2013; Elbracht et al., 2014; Hashiguchi et al., 2014; Manganelli et al., 2014; Berciano et al., 2015; 2016; Drew et al., 2015; Rebelo et al., 2016; Yang et al., 2016; Doppler et al., 2017; Horga et al., 2017; Jacquier et al., 2017; Nam et al., 2017; Bian et al., 2018; Fu and Yuan, 2018; Sainio et al., 2018; Ikenberg et al., 2019; Lerat et al., 2019; Machado et al., 2019; Aruta et al., 2021; Ando et al., 2022; Kim et al., 2022; Pipis et al., 2022; Chao et al., 2023; Petrucci et al., 2023).

The *NEFL* gene was the first causal gene to be identified in a dominant form of axonal CMT2 (CMT2E) (Mersiyanova et al., 2000). Since then, various mutations have been identified throughout the *NEFL* gene and more recently in the *NEFH* gene (Figure 6 with associated bibliography). Overall, NF-CMT accounts for 1% of diagnosed CMT cases and the majority of mutations are inherited through a dominant mode, although recessive forms and sporadic cases have been identified. As for many other CMT forms, NF-CMT have revealed a broader clinical picture than initially described. Thus, patients with NF mutations have shown to develop several defects within the central nervous system, including ataxia, hearing loss, dementia, nystagmus, tremor, schizophrenia, dysarthria, spasticity, and pain in addition to sensory-motor defects (Fabrizi et al., 2004; 2007; Züchner et al., 2004; Miltenberger-Miltenyi et al., 2007; Hashiguchi et al., 2014; Berciano et al., 2015; 2016; Doppler et al., 2017; Horga et al., 2017; Lerat et al., 2019; Machado et al., 2019; Kim et al., 2021; Chao et al., 2023).

As seen in Figure 6, the mutation types and positions differ greatly between the *NEFL* and *NEFH* genes.

Mutations in the *NEFL* gene have been identified in all domains of the protein: in the head, the ⍺-helical rod and in the tail (Figure 6A), domains which regulate various NF functions (see previous paragraph). Interestingly, the severity of the symptoms does not seem to correlate with the position of mutations (Stone et al., 2021), hence revealing an equal importance of all domains for NF functions.

So far, missense mutations are mostly inherited through a dominant mode and represent the most common CMT-causing mutations in the NEFL gene. Less frequently frameshift, in-frame deletion and nonsense mutations were identified. Interestingly, the latter mutations are only found in the rod domain where they segregate exclusively through a recessive mode of inheritance. Studies on the NFL^A367X^ mutant revealed that the premature termination of *NEFL* translation in both alleles abolishes NF-L expression in IPSC-MNs, due to the specific instability of its mRNA through a mechanism called non-sense mediated mRNA decay (Sainio et al., 2018). Astonishingly, a filamentous network positive for all other NF subunits could be seen in these cells (Sainio et al., 2018; 2021), findings which are surprising considering the current model presenting the necessity of the NF-L subunit for proper filament assembly. One could assume that in the IPSC-MN, α–internexin and peripherin may substitute NF-L in forming heteropolymers with NF-M and NF-H. Still, *in vivo*, this compensation does not seem to occur, as rare electron micrographs of patient biopsies evidence axons filled with MTs but with no filaments (*NEFL* ^E210X^ and *NEFL* ^E163X^) (Yum et al., 2009; Fu and Yuan, 2018)). A comprehensive analysis of all recessive nonsense mutations regarding the stability of the mRNA, the expression of NF-L in patient-derived cells, as well as the visualisation of the NF network in biopsies will be essential to further examine the requirement of NF-L in adult stages.

Another intriguing aspect is the clinical variability of the disease. Since the first mutation in the *NEFL* gene in an axonal form of CMT (CMT2E), others have been revealed in cases of demyelinating neuropathy (CMT1F) with reduced MNCV (Figure 6A). To make the picture even more complex, a variable clinical presentation (CMT1F and/or CMT2E and/or I-CMT) can result from distinct mutations on the same amino acid, or even from the same mutation (positions P8, P22, E90, N98, E396, P440). This is puzzling as NFs are confined to neuronal cells and not expressed in myelinating-Schwann cells. To explain this, several hypotheses could be proposed. An assumption would be that the demyelination (and reduced MNCV) may result from a general decrease in axonal caliber rather than axonal degeneration, as shown in the *NEFL* ^N98S/+^ knock-in model (Lancaster et al., 2018). In this context, all NF cases may correspond to axonal CMTs and would indicate a clinical variability due to gene modifiers or environmental factors. Alternatively, one could conceive that a crosstalk exists between the neuron and the Schwann cell, whereby dysfunctional NFs within the axons could signal to the glial cells. Finally, rare *in vivo* data have evidenced a transient induction of NF proteins in both early Schwann cells differentiation and dedifferentiation during Wallerian degeneration, which may reveal critical roles in myelin formation and repair after damage (Kelly et al., 1992; Roberson et al., 1992; Fabrizi et al., 1997; Yin et al., 2001). In patients, one could hypothesise that NF mutations may affect the functionality of the myelinating-Schwann cell, acting at the early stage of their maturation.

Mutations within the *NEFH* gene form an homogeneous group of dominant axonal CMT (CMT2CC) (Figure 6B), which differ from classical CMT due to their early and prominent involvement in proximal muscles and rapid decline of motor capacities (Aruta et al., 2021). The first cases (D1004Qfs*58 and P1008Afs*56) identified a novel type of mutation in NF-CMT (Rebelo et al., 2016): a frameshift variation that induces a loss of the terminating codon and the translation of short fragments in the 3′UTR of the *NEFH* mRNA. Predicted to be amyloidogenic, these extra fragments induce aggregation of the NF-H protein in neuronal cell lines. The identification of other mutations confirmed the homogeneity of the genetic alteration in CMT2CC and identifies a hot spot in the C-terminal portion of the tail domain of NF-H, that extends its length by < 60 amino acids.

### 3.3 Effects of NF mutants and roles in pathophysiology

Several laboratories have studied the effects of the *NEFL/H* mutations on filament assembly and cellular functions. Studies were mostly conducted *in vitro*, with only three mouse models generated for the NF-CMTs. Here, we will distinguish the findings evidenced *in vitro* and *in vivo*, and will discuss their relevance for NF biology and disease.

#### 3.3.1 In vitro studies evaluating self-assembly competence and integration into the NF network

In cells, studies on NF mutants were systematically conducted using overexpression, which makes sense for dominant forms. Accordingly, we will not discuss results obtained for the recessively inherited *NEFL* ^E210X^ mutation (with no NFL mutant in patient biopsy) and the *NEFL* ^T21Xfs^ found at heterozygous state (unclear if haplo-insufficient or dominant form). Various cellular systems have been used to determine the ability of single NF proteins (wild-type or mutants) to form a filament. To avoid bias of interpretation due to the presence of endogenous NF subunits (or IF types), the preferred cellular system is the SW13vim- human adrenal adenocarcinoma cells, which are deprived of IFs due to the spontaneous silencing of vimentin. An alternative yet rarely used IF-free system is the Sf9 insect cells. Once the ability of NF subunit to homopolymerise is established, research groups may combine other neuronal cell lines (CAD, SH-EP, NSC34, Neuro2a) and/or primary neurons with long neurites (cortical neuron, motor neuron, DRG sensory neuron).

Thus, the use of SW13- cells has helped demonstrate that only the human NF-L protein is able to homopolymerise, while the other cell types confirm the ability of wild-type NF-L to form filament in various conditions (Table 1 and associated bibliography). Indeed, when overexpressed, neither mouse nor rat NF-L can form a filamentous structure in the absence of other NF subunits (SW13vim- and Sf9 cells). Rodent NF-L was shown to form filament only as heteropolymer with another overexpressed NF subunit: NF-M and/or NF-H or ⍺-internexin in SW13vim‐ cells, or NF-M in Sf9 cells. Similarly, when IF proteins are endogenously expressed in SW13vim+ cells (vimentin), neuronal cell line and primary neurons (all NF subunits), the rodent NF-L protein is able to incorporate and colocalise with the endogenous network. Beautiful electron micrographs carried out using a quick-freeze deep-etching method in Sf9 cells revealed that co-expressed mouse NF-M and NF-L proteins co-assemble in parallelly arranged 10 nm filaments that form cross-bridges, similarly to what is seen in rat peripheral nerves (Nakagawa et al., 1995).

**TABLE 1:** Summary of assembly capacity of wild-type and CMT-mutated NF-L and NF-H proteins *in vitro*. In this table, we find the NF subunits overexpressed in different cellular systems. The capacity of wild type (WT) and mutant NF proteins to homopolymerise is determined in IF-free systems (SW13vim- or Sf9). Their effects on other IFs (exogenously or endogenously expressed) is shown in non-neuronal and neuronal cell types. Note that the study performed on the recessively inherited *NEFL* ^E210X^ mutation is not presented, as the patient has no detectable NF-L. Abbreviations: h, human; m, mouse; r, rat. Cell types used in the studies: SW13, a human adrenocarcinoma cell line that does not express vimentin (vim-) or expresses vimentin (vim+); Sf9, an insect clonal isolate of Spodoptera frugiperda Sf21 cells; Neuronal cell lines are: CAD (Cath. -a-differentiated), a mouse catecholaminergic neuronal tumour cell line; NSC34, a mouse motor neuron-like cell line; Neuro2a, a mouse neural crest-derived cell line; SH-EP, a human neuroblastoma cell line; primary neuronal cells are MNs, mouse primary motor neurons; DRG, mouse dorsal root ganglia sensory neurons.

		**Studied subunit**	**Cell type**	**Transfected subunit(s)**	**Endogenous subunit(s)**	**Phenotype**	**References**
**WT NF-L**	Human	WT hNF-L	SW13vim-	n/a	n/a	Filamentous network	Carter et al. (1998); Leung et al. (2006); Perez-Olle et al. (2002); Perez-Olle et al. (2005); Sasaki et al. (2006); Stone et al. (2019); Yum et al. (2009); Zhai et al. (2007)
		WT hNF-L	SW13vim-	WT NF-M and/or WT NF-H	n/a	Filamentous network (co-assembly with NF-M and/or NF-H)	Carter et al. (1998); Brownlees et al. (2002); Perez-Olle et al. (2002); Sasaki et al. (2006); Stone et al. (2019)
		WT hNF-L	SW13vim-	WT NF-M and Peripherin	n/a	Filamentous network (co-assembly with NF-M and Peripherin)	Stone et al. (2019)
		WT hNF-L	SW13vim+	n/a	Vim	Filamentous network (co-assembly with endogenous Vimentin)	Perez-Olle et al. (2002); Perez-Olle et al. (2004)
		WT hNF-L	CAD, SH-EP, NSC34, MNs	n/a	n/a	Filamentous network	Perez-Olle et al. (2005); Jacquier et al. (2017); Zhai et al. (2007)
		WT hNF-L	Cortical and DRG neurons	n/a	NF-M	Filamentous network (co-assembly with endogenous NF-M )	Brownlees et al. (2002); Sasaki et al. (2006)
		WT hNF-L	CAD	WT NF-M	n/a	Filamentous network (co-assembly with NF-M)	Perez-Olle et al. (2005)
	Rodent	WT r or mNF-L	SW13vim-	n/a	n/a	Diffuse pattern or puncta	Carter et al. (1998); Ching and Liem (1993); Gentil et al. (2012); Lee et al. (1993)
		WT r or mNF-L	SW13vim-	WT NF-M and/or NF-H or Internexin	n/a	Filamentous network (co-assembly with NF subunits)	Brownlees et al. (2002); Carter et al. (1998); Ching and Liem (1993); Lee et al. (1993); Perez-Olle et al. (2002); Tradewell et al. (2009); Yuan et al. (2009)
		WT mNF-L	Sf9	n/a	n/a	No filamentous network	Nakagawa et al. (1995)
		WT mNF-L	Sf9, N2a	WT NF-M	n/a	Filamentous network (co-assembly with NF-M in 10-nm filaments with cross bridges)	Nakagawa et al. (1995); Rao et al. (2023)
		WT mNF-L	SW13 vim+	n/a	Vim	Filamentous network (co-assembly with endogenous Vimentin)	Lee et al. (1993)
		WT mNF-L	MNs	n/a	n/a	Filamentous network	Gentil et al. (2012); Tradewell et al. (2009)
		WT mNF-L	Cortical neurons	n/a	n/a	Aggregation in the cell body	Yuan et al. (2009)
		WT mNF-L	Cortical neurons	WT NF-M or NF-H	n/a	Filamentous networks (co-assembly with endogenous NF-M or NF-H)	Yuan et al. (2009)
		WT rNF-L	Cortical neurons	WT NF-M	Internexin	Filamentous network (co-assembly with NF-M)	Stone et al. (2019)
**Mutant NF-L**	Human	hP8L, hP8R, hP8Q, hP22S, hP22T, hE89K, hN98S, hL268P, hQ332P, hE396K or P440L NF-L	SW13vim-	n/a	n/a	Aggregates or puncta	Perez-Olle et al. (2002); Perez-Olle et al. (2004); Perez-Olle et al. (2005); Sasaki et al. (2006); Stone et al. (2019); Yum et al. (2009)
		hT21Xfs NF-L	SW13vim-	n/a	n/a	Diffuse pattern	Leung et al. (2006)
		hT21Xfs NF-L	SW13vim-	WT NF-L	n/a	Diffuse pattern with short filament	Leung et al. (2006)
		hT21Xfs NF-L	SW13vim-	WT Internexin	n/a	Aggregates	Leung et al. (2006)
		hP8L, hP8R, hP8Q, hP22S, hP22T, hE89K, hN98S, hQ332P or hE396K NF-L	SW13vim-	WT NF-L or NF-M	n/a	Diffuse pattern, aggregates, puncta or inclusions	Perez-Olle et al. (2002); Perez-Olle et al. (2004); Perez-Olle et al. (2005); Stone et al. (2019); Yum et al. (2009)
		hP8R, hP8Q, hP22S, hL268P or hP440L NF-L	SW13vim-	WT NF-M	n/a	Filamentous network OR bundles	Perez-Olle et al. (2002); Perez-Olle et al. (2004); Perez-Olle et al. (2005); Stone et al. (2019)
		hP8R, hP22S, hP22T, hA148V or hQ332 NF-L	SW13vim-	WT NF-M and/or NF-H	n/a	Aggregates with WT NF-M and/or NF-H	Brownlees et al. (2002); Lee et al. (2008); Sasaki et al. (2006)
		hN98S, hQ332P or hE396K NF-L	SW13vim-	WT NF-M and Peripherin	n/a	Filamentous network	Stone et al. (2019)
		hP8Q, hP8R, hN98S, hQ332P NF-L	SW13vim+	n/a	Vim	Filamentous network OR bundles (co-assembly with endogenous Vimentin)	Perez-Olle et al. (2002); Perez-Olle et al. (2004)
		hP8L, hP8R, hE89K, hN98S or hQ332P NF-L	CAD	n/a	n/a	Aggregates in the soma and proximal segments	Perez-Olle et al. (2004); Perez-Olle et al. (2005)
		hP8R or hQ332P NF-L	CAD	WT NF-M	n/a	Aggregates in the soma and proximal segments	Perez-Olle et al. (2005)
		hP22S or hP22T NF-L	Cortical neurons	n/a	n/a	Aggregates in the soma	Sasaki et al. (2006)
		hP8R or hQ332P NF-L	Sympathetic neurons, cortical neurons, MNs, DRG neurons	n/a	NF-M	Aggregation in the soma and/or proximal neurites (with endogenous NF-M)	Brownless et al. (2002); Perez-Olle et al. (2005); Zhai et al. (2007)
	Rodent	rP8R or rQ332P NF-L	SW13vim-	WT NF-M and/or NF-H	n/a	Aggregates or puncta (with other subunits)	Brownlees et al. (2002); Perez-Olle et al. (2002)
		rP8R, rP22S, rN98S, rL269P, Q332P, rE396K or rP441L NF-L	Cortical neurons	WT NF-M	Internexin	Filamentous network (co-assembly with NF-M and Internexin)	Stone et al. (2019)
		mP8R or mQ332P	SW13vim-	n/a	n/a	Diffuse pattern	Gentil et al. (2012)
		mP8R NF-L	SW13vim-	WT NF-M	n/a	Filamentous network	Tradewell et al. (2009)
		mQ332P NF-L	SW13vim-	WT NF-M	n/a	Aggregates with NF-M (multiple inclusions)	Tradewell et al. (2009)
		mP8R NF-L	MNs	n/a	n/a	Filamentous network	Gentil et al. (2012)
		mP8R or mQ332P NF-L	MNs	n/a	n/a	Incorporation into filaments prior to aggregation	Tradewell et al. (2009)
**WT NF-H**	Human	WT hNF-H	SW13vim-	WT NF-L	n/a	Diffuse pattern	Bian et al. (2018)
		WT hNF-H	Neuro2a and SH-EP	n/a	n/a	Diffuse pattern	Jacquier et al. (2017); Rebelo et al. (2016)
		WT hNF-H	Neuro2a and SH-EP	WT NF-L	n/a	Filamentous network (co-assembly with NF-L)	Jacquier et al. (2017); Rebelo et al. (2016)
		WT hNF-H	MNs	n/a	n/a	Filamentous network	Jacquier et al. (2017)
	Rodent	WT rNF-H	SW13vim-	n/a	n/a	Puncta	Ching and Liem (1993)
		WT rNF-H	SW13vim-	WT NF-M or Internexin	n/a	Diffuse pattern or puncta	Ching and Liem (1993)
		WT mNF-H	SW13vim-	n/a	n/a	Diffuse pattern or puncta	Lee et al. (1993)
		WT mNF-H	SW13vim+	n/a	Vim	Diffuse pattern or puncta	Lee et al. (1993)
		WT mNF-H	Cortical neurons	n/a	n/a	Diffuse pattern	Yuan et al. (2009)
		WT mNF-H	Cortical neurons	NF-L	n/a	Filamentous network (co-assembly with NF-L)	Yuan et al. (2009)
**Mutant NF-H**	Human	hK1020Efs*43 NF-H	SW13vim-	WT NF-L	n/a	Aggregates	Bian et al. (2018)
		hK1003Rfs*59, hK1015Gfs*47, hD1004Qfs*58	SH-EP	n/a	n/a	Perinuclear aggregates	Jacquier et al. (2017)
		hK1003Rfs*59 NF-H	SH-EP	WT NF-L	n/a	Perinuclear aggregates with NF-L	Jacquier et al. (2017)
		hD1004Qfs*58 NF-H	Neuro2a	n/a	n/a	Perinuclear aggregates	Rebelo et al. (2016)
		hD1004Qfs*58 NF-H	Neuro2a	WT NF-L	n/a	Aggregates with NF-L	Rebelo et al. (2016)
		hK1003Rfs*59 or hK1015Gfs*47 NF-H	MNs	n/a	n/a	Perinuclear aggregates	Jacquier et al. (2017)

The effects of NF-L mutants (from human or rodent origin) have been extensively evaluated, mostly in SW13vim‐ cells, alone or in combination with one or two other NF subunits (Table 1). The detailed picture is complex but we can extract a few major points from these studies. Firstly, various NF-L mutants were shown to aggregate when overexpressed in SW13vim‐ cells, in the soma and proximal neurites in neurons. Secondly, the form of aggregates is highly variable, ranging from puncta, inclusions, small aggregates and large aggregate of various shapes. At this point, correlating a type of aggregate with specific mutations would be very difficult, considering the variability between studies and experimental conditions. Third, NF-L mutants seem to exert a dominant effect on other ectopically expressed wild-type subunits (alone or in combination) in SW13vim‐ cells and various neuronal types. Interestingly, this also applies to the endogenous network, as vimentin in SW13vim+ cells and endogenous NF-M in neurons were shown to colocalise with mutant NF-L. Nevertheless, effects vary greatly. Indeed, while in some cases other subunits co-aggregate with the mutant NF-L proteins, several reports show on the contrary that the other NF proteins (and vimentin) can protect to some degree the mutant NF-L from severe aggregation. For example, NFL^P8R^, NFL^P8Q^ and NFL^P22S^ form dense aggregates in SW13vim‐ cells, but are incorporated in an “apparent normal” network or bundle upon co-expression of NF-M, or together with endogenous vimentin filament in SW13vim+ cells (Perez-Olle et al., 2002; 2004; 2005; Tradewell et al., 2009). Other evidence shows that the mouse NFL^P8R^ and NFL^Q332P^ do not form aggregate in primary motor neurons at an early stage and have the capacity to be incorporated into the endogenous NF array (Gentil et al., 2012). Interestingly, a recent study has re-examined the capacity of human NF-L mutants to form aggregates in different cellular contexts expressing increasing NF content: the SW13vim‐ cells (no IFs), SW13vim- co-transfected with NF-M, SW13vim- co-transfected with NF-M + peripherin, primary cortical neurons co-transfected with NF-M (and containing endogenous NFs). Unexpectedly, none of the 7 human NF-L mutants aggregate in SW13vim‐ cells (Stone et al., 2019), as previously reported. Instead, they are able to form heteropolymers, with variable dependency on NF content (NF-M or NF-M + peripherin). In rat cortical neurons, rat NF-L mutants integrate into the endogenous NF array without forming any aggregate. Thus, with the examples cited above, these findings present the notion that while NF-L mutants may aggregate in certain contexts (possibly due to excessive overexpression or imbalance with other NF subunits), they are able to be incorporated into the filament as the wild-type protein. This different appreciation of the effects of *NEFL* mutations in dominant forms of CMT may reconcile with the rare data available from patients, which show more of an increased density and disorganised network than a defect in filament formation (Lus et al., 2003; Fabrizi et al., 2004; 2007; Doppler et al., 2017). Obtaining ultrastructural analysis of the NF array in patients and standardised biological systems to control protein (over)expression would be essential in order to compare and determine the effects of dominant mutations. Alternatively, a time-course study in motor neurons demonstrated that NFL^P8R^ and NFL^Q332P^ mutants could initially integrate into a pre-existing NF network and aggregate 7 days after overexpression, which may also indicate a long-term deleterious effect on NF assembly (Tradewell et al., 2009). Using the same mutants, another study reported that the mutants affect the oligomerisation process, possibly compromising filament formation (Gentil et al., 2013). Along with this, purified NF-L^P22S^ and NF-L^P22T^ are unable to form filament *in vitro*, even in the presence of wild-type NF-L (Sasaki et al., 2006), hence suggesting that NF-L mutants disrupt proper oligomer formation in cell-free systems.

Trying to conciliate data obtained from overexpressing systems *in vitro* and from patients is quite challenging. Still, the fact that the IPSC-MNs endogenously expressing the NFL^P8R^ mutant form distinct inclusions in the soma and neurites (Van Lent et al., 2021) clearly demonstrates that *NEFL* mutations can induce “aggregate” formation in a physiological context. Moreover, the detection of NFs as “aggregates” (using immunofluorescence) in the soma of DRG neurons from the knock-in NEFL^N98S/+^ mouse and as “packed/disorganised filaments” (using electron microscopy) (Zhao et al., 2017) demonstrates that endogenous NF-L mutants are seen differently depending on the level of analysis. Thus, we may suggest that NF mutants alter filament properties in various ways, and that what we visualise as “aggregates” in cells with low magnification methods may results from an abnormal arrangement of the filament.

Unlike the human NF-L protein, human NF-H itself is unable to form filament, as seen in SW13vim‐ cells, even in the presence of ectopic NF-L (Bian et al., 2018) (Table 1). In some studies, human NF-H was shown to be evenly distributed in the cytoplasm (Neuro2a, SH-EP), while others showed a filamentous network (SH-EP, Neuro2a) upon co-expression of NF-L in primary motor neurons. While these discrepancies may be explained by different times of analysis or expression levels, this clearly indicates that the human NF-H is self-assembly incompetent but can be incorporated to a pre-existing NF network. Rodent NF-H was shown to be unable to form filaments in any context, except in cortical neurons co-expressing NF-L (Table 1). Considering that purified NF-H is incapable of forming filament in assembly *in vitro* tests but does so in presence of purified NF-L (Geisler and Weber, 1981), more extensive analysis may be required to firmly document the capacity of NF-H to form heteropolymers in cells. Nevertheless, overexpression of several frameshift mutants of NF-H consistently revealed perinuclear aggregates, which trap ectopically expressed WT-NF-L, hence demonstrating the dominant effect conferred by the extra C-terminal fragment of NF-H.

#### 3.3.2 *In vitro* studies reveal defects in axonal transport and neuron physiology

The findings that NF mutants form aggregates in the soma or proximal segments in primary neurons (Brownlees et al., 2002; Perez-Olle et al., 2004; 2005; Zhai et al., 2007; Tradewell et al., 2009; Jacquier et al., 2017) or in IPSC-MNs (Van Lent et al., 2021) have led to the conclusion that their transport is reduced in disease. We could only find two studies directly addressing the effect of NF-L mutants on NF transport in rat cortical neurons. In the first study, the transport of EGFP-NF-M was reduced by about 50% in the context of aggregate-prone NFL^P8R^ and NFL^Q332P^ (Brownlees et al., 2002), while the other revealed an overall normal movement of various filament competent EGFP-NF-L mutants (Stone et al., 2019). While further studies will be needed to solve this issue, these findings suggest that the NF transport impairment in the context of aggregates may be caused by the alteration of NF integrity.

More literature is available on axonal transport, particularly of mitochondria. Thus, aberrant localisation, reduced length, reduced fusion, abnormal trafficking (reduced or increased), and decreased basal respiration were reported in neuronal cells expressing NFL^P8R^, NFL^P8L^, NFL^N98S^ and NFL^Q332P^ (Brownlees et al., 2002; Perez-Olle et al., 2004; 2005; Tradewell et al., 2009; Gentil et al., 2012; Saporta et al., 2015), and also in IPSC-MN with *NEFL* ^P8R^ mutation (Van Lent et al., 2021). However, no alteration of mitochondria dynamics was evidenced in human motor neuron spheroids with the NFL^N98S^ mutant (Maciel et al., 2020). Other dysfunctions, such as Golgi fragmentation and lysosomal motility were occasionally reported for NF-L mutants (Perez-Olle et al., 2005; Van Lent et al., 2021). Rare studies have evidenced electrophysiological and neuronal dysfunctions for *NEFL* mutations. Thus, reduced action potential threshold was reported in IPSC-MN with *NEFL* ^N98S^ mutation (Saporta et al., 2015); reduced neurite length and hyperconnectivity were evidenced in IPSC-MN with *NEFL* ^P8R^ mutation (Van Lent et al., 2021), as well as apoptosis in SH-EP and spinal cord motor neurons overexpressing NF-H mutant (Jacquier et al., 2017) and progressive neurotoxicity in motor neurons overexpressing NFL^P8R^ and NFL^Q332P^ (Zhai et al., 2007). In apparent normal NF networks, neurons deprived of NF-L (as seen in the recessive forms of NF-CMT) also showed interesting abnormalities. Thus, IPSC-MN with *NEFL* ^A366X^ (or CRISPR-KO cells) exhibit increased mitochondria trafficking and reduced amplitude of miniature excitatory postsynaptic currents (Sainio et al., 2021).

Overall, studies predominantly investigated mitochondria dynamics, but the current knowledge regarding the physiological mechanisms underlying the NF-CMT pathology is rather limited. Efforts initiated to investigate neuronal (electro)physiology in cellular systems would be of great benefit to the field, in combination with the study of NF-CMT in animal models.

#### 3.3.3 *In vivo* models of NF-CMT

To date, only three animal models have been generated for NF-L CMT disease. Here we will not discuss the transient expression of NF-H mutant in animals, which only confirmed aggregation and caspase-3 activation in chick embryos (Jacquier et al., 2017) and revealed shorter motor axons in zebrafish (Rebelo et al., 2016). It is worth noting that one transgenic mouse model overexpressing a mouse *NEFL* ^L394P^ mutation not identified in CMT disease showed specific alterations of motor neurons, with abnormal NF accumulation in the soma and axonal degeneration (Lee et al., 1994).

Two transgenic models were created, overexpressing the human *NEFL* E397K (*hNEFL* ^E397K^ (Shen et al., 2011)) or the P22S transgene (*hNEFL* ^P22S^ (Dequen et al., 2010)), the latter in an inducible manner using the Tet-off tetracycline-regulated system.

From 4 months of age, *hNEFL* ^E397K^ mice exhibit abnormal gait (Dale et al., 2012), sensory defects (reduced sensitivity to mechanical stimuli) (Villalón et al., 2017), motor deficits (reduced locomotion, reduced motor coordination), aberrant limb posture, reduced MNCV and muscle atrophy of the lower limbs, without denervation (Shen et al., 2011). The total number of motor and sensory axons is not affected but a shift towards a decrease in large caliber axons is observed. Regarding NFs, there was no sign of NF aggregation or impaired disorganisation/density in *hNEFL* ^E397K^ mice, only increased NF phosphorylation in the soma of spinal motor neuron (Shen et al., 2011). Detailed analysis of muscle spindle morphology revealed a reduced area at pre-symptomatic stage, suggesting proprioceptive sensory deficits in the *hNEFL* ^E397K^ model (Villalón et al., 2017).

The *hNEFL* ^P22S^ mice exhibit from 6 months of age aberrant limb posture, abnormal gait, learning defects, motor dysfunctions (reduced coordination), but in contrast to the *hNEFL* ^E397K^ mice, the model presents no sensory deficits (normal mechanical response) and present muscle hypertrophy with signs of denervation (Dequen et al., 2010; Filali et al., 2011). As for the other model, the *hNEFL* ^P22S^ mice do not present NF alterations (except for rare NF-H positive inclusions in spinal motor neurons) nor neuronal degeneration. Interestingly, the removal of the mutant NF-L transgene (following 3 months of treatment with doxycycline) ameliorates several motor abnormalities, attenuates muscle hypertrophy and restores muscle innervation (Dequen et al., 2010).

Overall, although both CMT models present symptoms of the sensory-motor system, none of the two transgenic mouse models exhibit overt neurodegeneration and NF alterations, and only mild signs of nerve pathology were reported.

A third *NEFL* ^N98S/+^ knock-in mouse model was generated by the introduction of the CMT mutation at the endogenous locus. This mouse model presents abnormal hindlimb posture, develops tremor from 4 weeks of age (Adebola et al., 2015), and subsequently exhibits altered balance/gait from 6 weeks of age (Lancaster et al., 2018). Mobility and sensitivity to thermal stimuli remain unaltered. Interestingly, an abnormal distribution of NF-L was observed in different parts of the nervous system (spinal cord, DRG neurons, cerebellum) at pre-symptomatic stage (post-natal day 7) and persisted during aging (18 months of age). Thus, accumulation of NF-L was detected in the soma of neurons, while a decrease was observed in the axonal tracts. In the sciatic nerves, only a few NFs could be detected upon ultrastructural examination (12 months of age) and, as expected from the literature, increased MT density and decreased axonal caliber were detected (Adebola et al., 2015; Lancaster et al., 2018). Early analysis (from 8 weeks of age) of various regions of the nervous system (optic nerves, spinal cord, femoral/sciatic/caudal nerves) evidenced fewer NFs, smaller myelinated axons and reported a reduction of both the amplitude and NCV in the caudal nerves (Lancaster et al., 2018). In contrary, cell bodies and proximal axons of the sensory-motor system exhibit NF accumulation and are disorganised. Another study revealed NF aggregation in the soma of DRG neurons in adult *NEFL* ^N98S/+^ mice and showed that the NF-L mutant switches the soluble/insoluble balance towards insolubility in tissue (Zhao et al., 2017). Overall, these findings suggest that the *NEFL* ^N98S^ mutant has a dominant effect on NF architecture, most likely trapping and disorganising NFs within the soma. While the phenotype is milder compared to human CMT disease, the *NEFL* ^N98S/+^ mouse is the first model to present NF abnormalities and signs of axonal degeneration and regeneration.

Interestingly, several *in vivo* studies have revealed the essential role of NFs in promoting axonal regeneration. Indeed, the *hNEFL* ^E397K^ mice show reduced recovery from nerve crush injury on sciatic nerves, which exacerbates the neuropathy: increased mechanical allodynia, premature gait dysfunction and disrupted limb coordination (Villalón et al., 2015). Reduced regeneration was also reported in two mouse models depleted of axonal NFs, *NEFL* ^−/−^ (Zhu et al., 1997) and *NEFH* ^LACZ^ mice (Cassereau et al., 2013), in which NFs were shown to accumulate in the soma.

#### 3.3.4 Current therapeutic approaches toward NF-CMT

To date, there is no treatment for any form of the NFs-CMT disease. However, several therapeutic strategies aimed at enhancing expression of wild-type NF-L in recessive forms, inactivating mutated NF-L and restoring normal NF organisation have been investigated. Other approaches targeting the downstream cellular alterations are also undergoing.

In recessive forms of NF-CMT, heterozygous carriers were reported to be asymptomatic, suggesting that a single functional NEFL allele is sufficient for normal neurological function (Abe et al., 2009; Yum et al., 2009; Fu and Yuan, 2018). Thus, drugs that induce translational read-through or inhibit non-sense mediated mRNA decay were tested in IPSC-MNs carrying the *NEFL* ^A367X^ homozygous mutation. While this first attempt failed to show an increase of NF-L level (due to neurotoxicity) (Sainio et al., 2021), this avenue is certainly worth further investigation. In another IPSC-MN model for the dominant *NEFL* ^N98S^ mutation, authors used the CRISPR/cas9 technology to correct the mutation (*NEFL* ^N98S−cor^) or specifically silence the N98S allele (*NEFL* ^N98S−fs^) by introducing a frameshift in the sequence (Feliciano et al., 2021). This strategy induces a 42% reduction of NF-L^N98S−fs^ expression in the IPSC-MN, due to the specific decrease of the *NEFL* mutant transcript. Interestingly, gene editing restored normal intensity of NFs in the soma and electrical activity in both the *NEFL* ^N98S−cor^ and *NEFL* ^N98S−fs^ MNs, hence representing a promising therapeutic avenue for dominant missense mutations in the *NEFL* gene. Another interesting approach, initiated on IPSC-MNs is based on the amelioration of the mitochondrial-associated defects in CMT2, as shown by the benefits induced by MAP3K12 inhibitors on mitochondrial morphology and basal respiration in *NEFL* ^LP8R^ human MNs (Van Lent et al., 2021).

Three dimensional spheroids were recently developed from IPSC-MNs of NF-CMT2 patients. *NEFL* ^N98S^ spheroids revealed abnormal NF distribution along the axons (10-fold increase of NF deposits without change in abundance), phenotype which is partially reversed by two kinase inhibitors (PLKi and CK2) known to phosphorylate NFs (Maciel et al., 2020). The role of phosphorylation in regulating various aspects of NF dynamics has been exploited as a possible therapeutic strategy for NF-CMT. In particular, the phosphorylation of the head domain of NFs is known to induce polymer disassembly. The findings demonstrating that purified NF-L^P22S^ and NF-L^P22T^ proteins are less phosphorylated by ERK and cdk5 kinases (Sasaki et al., 2006) led to the proposal that their aggregation may result from an excessive stability of the proteins. Thus, increasing PKA-dependent phosphorylation (acting on other residues in the head) was shown to increase the solubility of NF-L mutants and to partially dissolve NF aggregates in cortical neurons (Sasaki et al., 2006). Other alternative approaches aiming at destabilising NFs would be to modulate regulatory factors in disease. In this direction, the 14-3-3 protein, which was shown to interact with the NF-L head domain in a phospho-dependent manner induces NF disassembly *in vitro* and reduces aggregation caused by NF-L^P8L^, NF-L^P22S^ and NF-L^P22T^ mutants in SW13vim-cells NFs (Miao et al., 2013).

Amongst therapeutic strategies initiated for NF-CMT, there is a particular focus on molecular chaperones which protect cells from misfolded proteins. This interest merged from the findings that mutations in the small heat-shock protein 27 (HSP27 also called HSPB1) are causal for the dominant axonal CMT2F (Evgrafov et al., 2004) and induce aggregation when overexpressed in SW13vim-cells, NSC34 cells and cortical neurons (Evgrafov et al., 2004; Ackerley et al., 2006; Zhai et al., 2007). Studies showed that the wild-type HSPB1 associates with NF proteins preferentially in a polymerising state to interfere with it and increase the pool of soluble NF particles (Nefedova et al., 2017). This study indicates that HSPB1 modulates the transition between tetramers and filament and suggests that its mutation in CMT2F would cause an excessive stabilisation of the wild-type NF (leading to NF aggregation). It also suggests that HSPB1 could be used to destabilise NF aggregates in CMT2E. Thus, overexpression of HSPB1 was shown to decrease aggregation induced by the NF-L^P8R^ and NF-L^Q332P^ mutants in SW13vim‐ cells (Zhai et al., 2007). Importantly, similar results were obtained in primary motor neurons transfected with the NF-L^P8R^ mutant, resulting in an improvement of cell viability, hence representing a substantial beneficial outcome for therapeutic efficacy. In addition, the fact that the neurotoxicity of mutant HSPB1 on motor neurons is almost eradicated in *NEFL* ^−/−^ motor neurons (Zhai et al., 2007) indicates that NFs represent a major target for therapeutic development for various forms of CMTs. Intriguingly, heat shock proteins exhibit a specificity toward NF-L mutants, with HSPB1 being preferentially effective on NF-L^P8R^ but not NF-L^Q332P^ and HSPA1 only on NF-L^Q332P^ in MNs (Tradewell et al., 2009). This implies that the efficacy of therapy using chaperone or chaperone inducers must carefully examine the type of chaperone that would be effective on given NF-L mutants. Treatment with celastrol, an inducer of HSPA1 in motor neuron is only able to decrease NF-L^Q332P^ inclusions and not NF-L^P8R^ ones (Gentil et al., 2013). As celastrol exhibits a cell type specific mode of action (induction of HSPA1 specifically in motor neurons, HSPB1 specifically in sensory neurons), its benefit in treating a sensory motor neuropathy appears to be rather limited. Nevertheless, these studies have opened the avenue for the identification of chaperone inducers as a therapeutic strategy for NF-CMT.

Noteworthy, HSPB1 has also been shown to control NF transport (through regulation of NF binding to kinesin via Cdk5-mediated phosphorylation of NFs) (Holmgren et al., 2013), MT stability (Almeida-Souza et al., 2011) (by binding to tubulin) and autophagy flux (through interaction with the autophagy receptor p62) (Haidar et al., 2019), which may suggest multiple functions of HSPB1 on NFs and may therefore reveal potential downstream targets for therapeutic intervention in NF-CMTs (Vendredy et al., 2020).

## 4 Concluding remarks and future directions

Neurofilaments are composed of four subunits, whose stoichiometry varies between neuronal types during development and aging. With a general mode of assembly akin to IFs, NFs are expected to exhibit a high heterogeneity, with NF-L (and to a lesser extent ⍺-internexin/peripherin) as a core protein. One challenge ahead will be to scrutinise the molecular and structural diversity of NFs and to determine how this contributes to cell signalling. NFs have been shown to play key physiological roles in protecting axonal architecture, controlling nerve conduction and neurotransmission and regulating cytoskeletal dynamics. Considering the broad clinical picture of NF-CMT forms in the CNS and the identification of NF mutations in other neurological diseases, the field will certainly uncover additional unknown fundamental roles of NFs in various parts of the nervous system. Another interesting aspect to consider in the future is the mechanisms whereby NFs respond to neuronal needs within the nervous system. With a basal stationary NF network that provides the structural scaffold of the axon, the neuron has established a sophisticated set of NF regulators to provide, at a minimal energy cost, the dynamics necessary to fulfil local demands and respond to normal stimuli and injury. While our knowledge on NFs has considerably increased in the last few decades, the transport and turn-over of NFs are key determinants of NF dynamics that require further investigation. A major challenge ahead is to apprehend with a spatial and temporal resolution the various elements that regulate NF dynamics and signalling. While manipulating NF stoichiometry continues to be an essential approach in tackling these key questions, a deep investigation of the various NF diseases using IPSC, organoids and animal models is absolutely required to expand our knowledge on NF biology and its physiological relevance. Thus, the study of recessive NF-CMT forms may suggest a temporal control for the requirement of specific core proteins in filament assembly. Conflicting outcomes obtained on dominant CMT forms are challenging our view on what is an aggregate and how NF mutants affect filament assembly and structure. In the future, understanding the yet unknown mechanisms underlying neurodegeneration in disease is a main biomedical challenge that will fuel discoveries on the physiological roles of NFs, therapeutic innovation in NF-CMTs, and more widely in most neurodegenerative diseases.
